# The genomic history of the Middle East

**DOI:** 10.1016/j.cell.2021.07.013

**Published:** 2021-09-02

**Authors:** Mohamed A. Almarri, Marc Haber, Reem A. Lootah, Pille Hallast, Saeed Al Turki, Hilary C. Martin, Yali Xue, Chris Tyler-Smith

**Affiliations:** 1 Wellcome Sanger Institute, Wellcome Genome Campus, Hinxton CB10 1SA, UK; 2 Department of Forensic Science and Criminology, Dubai Police GHQ, Dubai, United Arab Emirates; 3 Institute of Cancer and Genomic Sciences, University of Birmingham, Birmingham B15 2TT, UK; 4 Centre for Computational Biology, University of Birmingham, Birmingham B15 2TT, UK; 5 Institute of Biomedicine and Translational Medicine, University of Tartu, Tartu 50411, Estonia; 6 Translational Pathology, Department of Pathology and Laboratory Medicine, King Abdulaziz Medical City, Ministry of National Guard-Health Affairs, Riyadh, Saudi Arabia; 7 Department of Genetics & Genomics, College of Medicine and Health Sciences, United Arab Emirates University, Al Ain, United Arab Emirates

**Keywords:** Arabia, Levant, Selection, Basal Eurasian, Near East, Aridification, Climate change, Neanderthal, Population genetics, Migration

## Abstract

The Middle East region is important to understand human evolution and migrations but is underrepresented in genomic studies. Here, we generated 137 high-coverage physically phased genome sequences from eight Middle Eastern populations using linked-read sequencing. We found no genetic traces of early expansions out-of-Africa in present-day populations but found Arabians have elevated Basal Eurasian ancestry that dilutes their Neanderthal ancestry. Population sizes within the region started diverging 15–20 kya, when Levantines expanded while Arabians maintained smaller populations that derived ancestry from local hunter-gatherers. Arabians suffered a population bottleneck around the aridification of Arabia 6 kya, while Levantines had a distinct bottleneck overlapping the 4.2 kya aridification event. We found an association between movement and admixture of populations in the region and the spread of Semitic languages. Finally, we identify variants that show evidence of selection, including polygenic selection. Our results provide detailed insights into the genomic and selective histories of the Middle East.

## Highlights

• Middle Easterners do not have ancestry from an early out-of-Africa expansion
• Basal Eurasian and African ancestry in Arabians deplete their Neanderthal ancestry
• Populations experienced bottlenecks overlapping aridification events
• Identification of recent single and polygenic signals of selection in Arabia

## Graphical abstract

**Figure:**
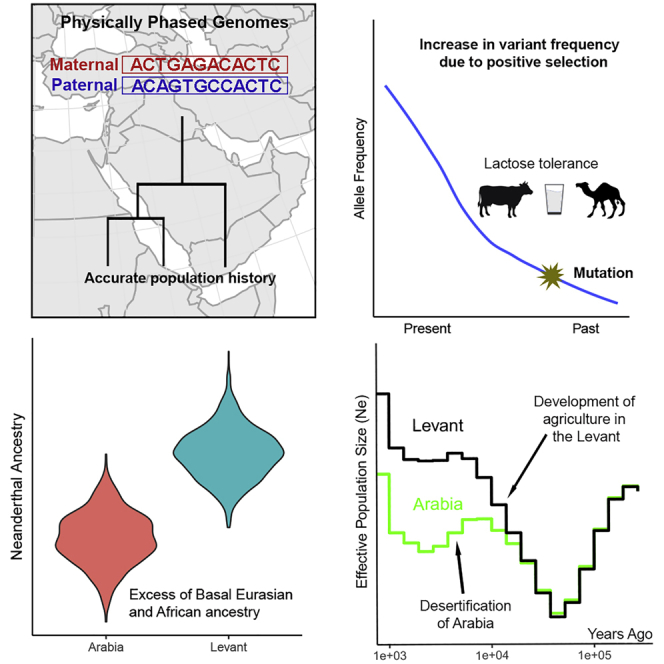

## Teaser

A high-coverage resource of physically phased genomes from eight Middle Eastern populations generated via linked-read sequencing provides insights into a genetically understudied region and enables more comprehensive study of population history and the detection of millions of variants common to the Middle East but outside short-read accessibility masks and not previously cataloged. It enhances our understanding of regional ancestry, the spread of languages, the effects of climate change on populations, and the evolutionary history of genetic variants.

## Introduction

Global whole-genome sequencing projects have provided insights into human diversity, dispersals, and past admixture events (Bergström et al., 2020; Mallick et al., 2016; GenomeAsia100K Consortium, 2019; 1000 Genomes Project Consortium et al., 2015). However, many populations remain understudied, which restricts our understanding of genetic variation and population history and may exacerbate health inequalities (Sirugo et al., 2019). A region particularly understudied by large-scale sequencing projects is the Middle East (Fernandes et al., 2019; Abou Tayoun and Rehm, 2020). Situated between Africa, Europe, and South Asia, it forms an important region to understand human evolution, history, and migrations. The region contains some of the earliest evidence of modern humans outside Africa, with fossils dated to at least 177 thousand years ago (kya) and ∼85 kya identified in the Levant and North West Arabia, respectively (Hershkovitz et al., 2018; Groucutt et al., 2018). In addition, tools and footprints attributed to modern humans have also been identified in Arabia ∼120 kya (Armitage et al., 2011; Stewart et al., 2020). Although most of Arabia is a hyper-arid desert today, there were several humid periods resulting in a “green Arabia” in the past which facilitated human dispersals, with the onset of the current desert climate thought to have started around 6 kya (Petraglia et al., 2020). The toggling from humid to arid periods in the late Pleistocene and Holocene has been proposed to result in population movements adapting to the climate. The Neolithic transition within Arabia may have developed independently within the region or resulted from an expansion of Levantine Neolithic farmers southward (Drechsler, 2009; Uerpmann et al., 2010; Crassard and Drechsler, 2013a, 2013b; Hilbert et al., 2015). To address such questions, we generated and analyzed a high-coverage physically phased dataset of populations from the Arabian Peninsula, the Levant, and Iraq. In addition to creating a catalog of genetic variation in an understudied region that will assist future medical studies, we have investigated the population structure, demographic and selective histories, and admixture events with modern and archaic humans.

## Results

### Dataset and sample sequencing

**Figure 1:**
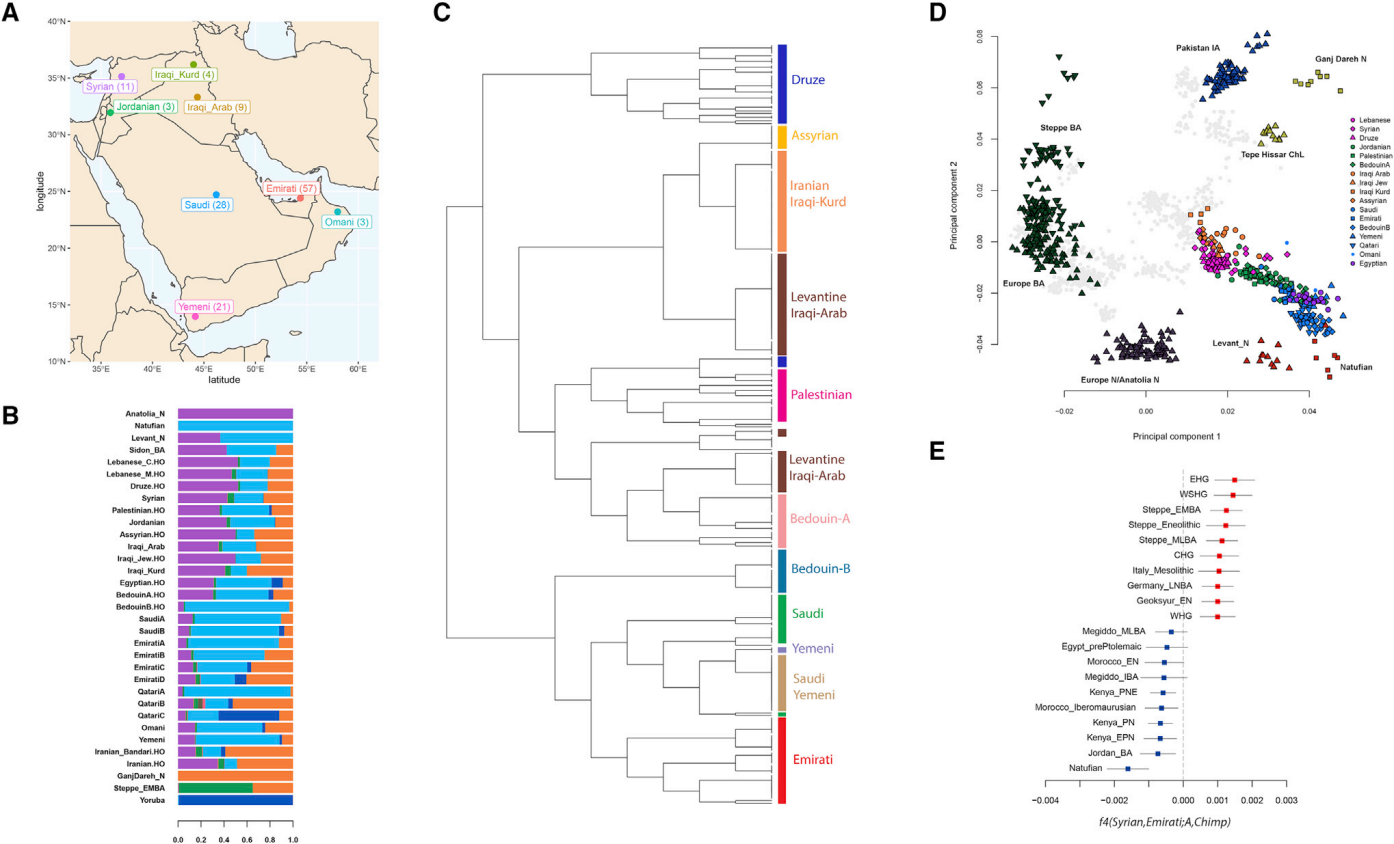
Overview of the dataset and population structure of the Middle East (A) Map illustrating the populations sampled in this study, with number of individuals shown in brackets. We use the term “Arabian” in this study to refer to samples from the Arabian Peninsula (Emirati, Saudi, and Yemeni), Levantine for Syrians and Jordanians, and Iraqi-Arabs and Iraqi-Kurds for samples from Iraq. (B) Temporally aware model-based clustering using ~88,000 transversions and 9 time points, showing K = 8 when the Anatolia_N and Natufian components split. “.HO” suffix refers to samples from the Human Origins Dataset. (C) fineSTRUCTURE tree of modern-day Middle Easterners with population clusters highlighted. (D) Principal component analysis of ancient and modern populations. Eigenvectors were inferred with present-day populations from the Middle East, Europe, and Central and South Asia. The ancient samples were then projected onto the plot (all modern non-Middle Easterners shown as gray points). See Figure S1 for more details. (E) Genetic contrast between the Levant and Arabia illustrated using the statistic *f4*(Syrian,EmiratiA;Ancient,Chimpanzee) and ± 3 standard errors with the 10 lowest (blue) and 10 highest (red) f4-stats.

We sequenced 137 whole genomes from eight Middle Eastern populations (Figure 1A) to an average coverage of 32× using a library preparation method that preserves long-range information from short reads and aligned them to the GRCh38 reference (STAR Methods). An advantage of using this “linked-read” technology is the reconstruction of physically phased haplotypes and improved alignments at repetitive regions that confound short-read aligners. All populations investigated speak Arabic, a Semitic language of the Afro-Asiatic language family, with the exception of the Iraqi Kurdish group who speak Kurdish, an Iranian language belonging to the Indo-European family. After quality control (STAR Methods), we identified 23.1 million single-nucleotide variants (SNVs). We compared our dataset to variants identified in the recently released Human Genome Diversity Project (HGDP-CEPH) study (Bergström et al., 2020). We found 4.8 million autosomal SNVs in our dataset that are not found in the HGDP. As expected, most of the these variants are rare (93%, <1% minor allele frequency); however, ∼370,000 are common (>1%). Interestingly, most of these common variants are outside the accessibility mask defined by Bergström et al., 2020 (∼246,000 variants in ∼27% of the genome). This illustrates the importance of sequencing genetically under-represented populations such as Middle Easterners and the inclusion of regional-private variants in future medical studies. It also demonstrates that a significant amount of unknown variation resides in regions that are not accessible to standard short-reads.

### Population structure and admixture

Uncovering population structure and past admixture events is important for understanding population history and for designing and interpreting medical studies. We explored the structure and diversity of our dataset using both single-variant and haplotype-based methods. After merging our dataset with global populations, fineSTRUCTURE (Lawson et al., 2012) identified genetic clusters that are concordant with geography and showed that self-labeled populations generally formed distinct clusters (Figure 1C). Populations from the Levant and Iraq (Lebanese, Syrians, Jordanians, Druze, BedouinA, and Iraqi-Arabs) clustered together, while Iraqi-Kurds clustered with Central Iranian populations. Arabian populations (EmiratiA, Saudis, Yemenis, and Omanis) clustered with BedouinB from the HGDP. Within the Emirati population, we identified subpopulations with excess Iranian and South Asian ancestries (EmiratiB and EmiratiC; Figure 1B). We also found subpopulations harboring relatively higher African ancestry (SaudiB, EmiratiD; Figure 1B).

**Figure S1:**
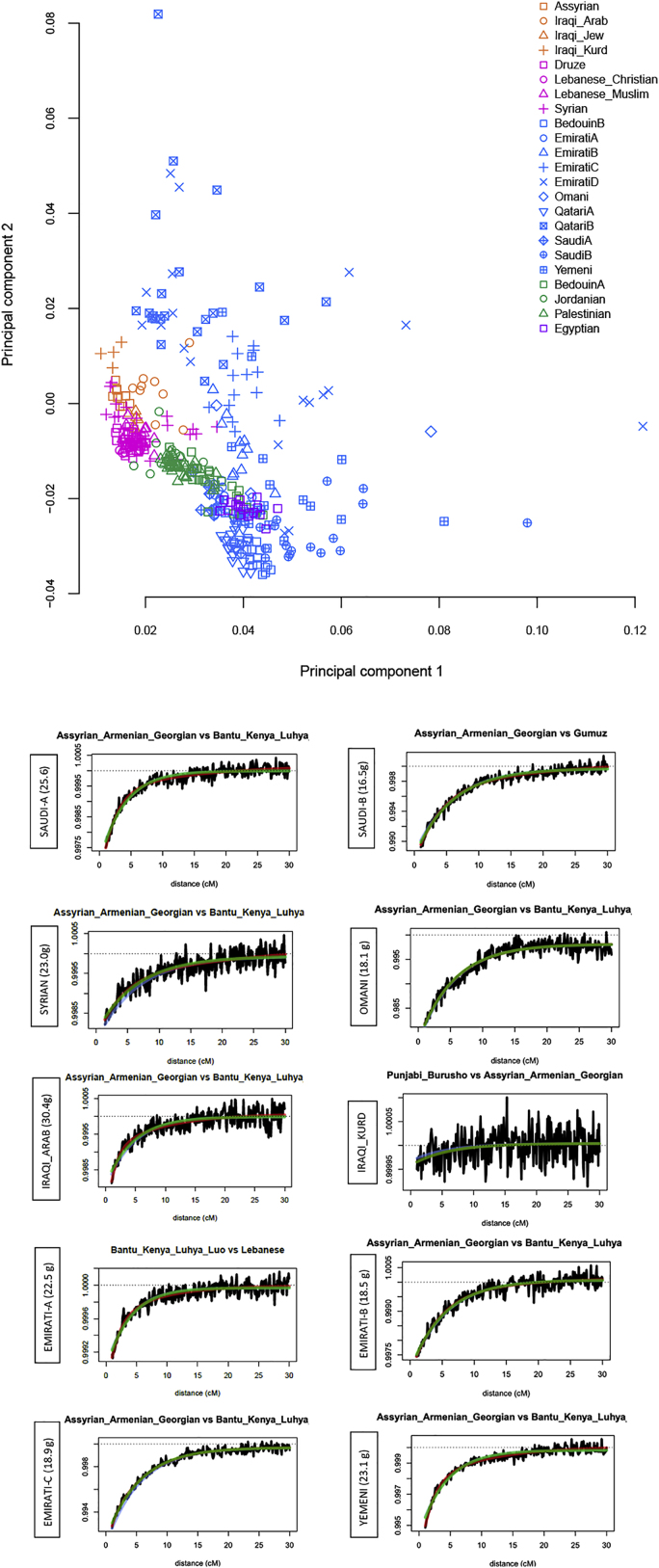
Population structure and admixture, related to Figure 1 **Top:** Principal component analysis. Plot similar to Figure 1D but magnifying the modern Middle Eastern cluster and also including other subpopulations (e.g., EmiratiB and QatariB). **Bottom**: Testing for recent admixture using modern population as sources with GLOBETROTTER. Co-ancestry curves showing relative probability of jointly copying two chunks from donors at varying genetic distances. The curves fit an exponential decay (1-date green line, 2-date red line). The positive slope implies that these donors represent potential proxies to the admixing sources. The estimated admixture date is illustrated on the left of each figure, g for generations. We find that the two putative sources are always a Middle Eastern and an East African population. The dates are in general agreement with MALDER (Table S1). The Iraqi_Kurds are notable for not showing evidence of recent admixture.

We next analyzed our samples in the context of ancient regional and global populations. Principal component analysis (Figures 1D and S1) shows that present-day Middle Easterners are positioned between ancient Levantine hunter-gatherers (Natufians), Neolithic Levantines (Levant_N), Bronze Age Europeans, and ancient Iranians. Arabians and Bedouins are positioned close to ancient Levantines, while present-day Levantines are drawn toward Bronze Age Europeans. Iraqi-Arabs, Iraqi-Kurds, and Assyrians appear relatively closer to ancient Iranians. We found that most present-day Middle Easterners can be modeled as deriving their ancestry from four ancient populations (Table 1): Levant_N, Neolithic Iranians (GanjDareh_N), Eastern Hunter Gatherers (EHG), and an ∼4,500-year-old East African (Mota). We observed a contrast between the Levant and Arabia: Levantines have excess EHG ancestry (Figure 1E), which we showed previously had arrived in the Levant after the Bronze Age along with people carrying ancient south-east European and Anatolian ancestry (Haber et al., 2017, 2020). Our results here show this ancestry is much higher in the Levant compared to Arabia (Table 1). Another contrast between the Levant and Arabia is the excess of African ancestry in Arabian populations. We found that the closest source of African ancestry for most populations in our dataset is Bantu Speakers from Kenya, in addition to contributions from Nilo-Saharan speakers from Ethiopia. We estimate that African admixture in the Middle East occurred within the last 2,000 years, with most populations showing signals of admixture around 500–1,000 years ago (Figure S1; Table S1), in agreement with previous studies (Hellenthal et al., 2014).

**Table 1:** Modeling present-day Middle Easterners as deriving their ancestry from four ancient populations using qpAdm

Test	P value for rank = 3	Levant_N	Iran_N	EHG	Mota	P value for rank = 3	Natufian	Iran_N	EHG	Mota
Assyrian.HO	2.85E-03	0.32	0.60	0.10	−0.02	2.40E-05	0.39	0.55	0.09	−0.03
BedouinA.HO	1.55E-01^a^	0.42	0.40	0.09	0.09	6.59E-04	0.48	0.36	0.09	0.07
BedouinB.HO	6.03E-01^a^	0.54	0.35	0.06	0.05	2.45E-02	0.57	0.33	0.07	0.04
Druze.HO	9.42E-03	0.38	0.48	0.14	0.002	1.00E-05	0.46	0.44	0.12	−0.01
Egyptian.HO	1.85E-01^a^	0.45	0.33	0.08	0.15	6.18E-03	0.50	0.31	0.07	0.12
EmiratiA	1.33E-02	0.50	0.42	0.06	0.03	3.09E-01^a^	0.53	0.39	0.07	0.02
EmiratiB	4.19E-04	0.40	0.50	0.07	0.04	1.39E-02	0.47	0.44	0.07	0.02
EmiratiC	2.00E-06	0.30	0.54	0.09	0.08	2.19E-03	0.35	0.49	0.09	0.07
EmiratiD	9.00E-06	0.21	0.55	0.11	0.14	1.94E-03	0.26	0.51	0.10	0.13
Iranian.HO	2.22E-03	0.19	0.69	0.13	−0.01	2.54E-03	0.26	0.64	0.12	−0.02
Iranian_Bandari.HO	0.00E+00	0.11	0.71	0.12	0.06	6.00E-06	0.15	0.68	0.12	0.06
Iranian_Jew.HO	4.10E-02	0.32	0.56	0.13	−0.01	2.03E-03	0.40	0.51	0.11	−0.02
Iraqi_Arab	8.83E-02^a^	0.31	0.54	0.13	0.03	3.94E-02	0.39	0.49	0.11	0.01
Iraqi_Jew.HO	1.09E-02	0.35	0.55	0.11	−0.01	1.16E-03	0.42	0.50	0.09	−0.02
Iraqi_Kurd	7.30E-02^a^	0.24	0.62	0.16	−0.01	4.98E-02	0.32	0.56	0.14	−0.02
Jordanian	1.14E-01^a^	0.43	0.43	0.13	0.02	6.60E-03	0.50	0.39	0.11	0.003
Jordanian.HO	2.14E-01	0.37	0.43	0.14	0.06	1.07E-02	0.47	0.38	0.11	0.04
Lebanese_Christian.HO	2.77E-02	0.41	0.46	0.13	−0.004	7.10E-05	0.49	0.42	0.11	−0.02
Lebanese_Muslim.HO	1.19E-01^a^	0.38	0.49	0.12	0.02	5.33E-04	0.45	0.44	0.11	0.001
Omani	2.99E-01^a^	0.40	0.40	0.10	0.10	4.69E-02	0.46	0.37	0.09	0.08
Palestinian.HO	2.41E-02	0.40	0.43	0.11	0.06	3.88E-04	0.48	0.39	0.10	0.04
QatariC	2.35E-02	0.16	0.18	0.04	0.62	1.34E-03	0.16	0.20	0.05	0.60
QatariA	1.10E-01^a^	0.58	0.35	0.04	0.02	1.22E-01^a^	0.59	0.34	0.06	0.01
QatariB	0.00E+00	0.16	0.69	0.11	0.05	0.00E+00	0.21	0.64	0.11	0.04
Saudi.HO	2.17E-01^a^	0.50	0.40	0.07	0.04	7.59E-02^a^	0.52	0.38	0.08	0.02
SaudiA	6.80E-02^a^	0.50	0.42	0.06	0.03	1.51E-01^a^	0.53	0.39	0.07	0.02
SaudiB	1.37E-01^a^	0.51	0.32	0.05	0.12	3.73E-02	0.52	0.31	0.07	0.10
Syrian	1.12E-01^a^	0.34	0.50	0.15	0.02	1.40E-02	0.41	0.46	0.13	0.01
Syrian.HO	1.63E-02	0.37	0.45	0.12	0.05	2.80E-05	0.44	0.42	0.11	0.04
Yemeni	1.88E-02	0.52	0.35	0.05	0.09	6.41E-02^a^	0.55	0.33	0.05	0.07
Yemeni.HO	4.23E-02	0.38	0.40	0.06	0.16	9.64E-02^a^	0.42	0.38	0.07	0.14

The choice of a 4-way admixture derives from previous knowledge on the region with Levant_N and GanjDareh_N contributing significant ancestry to ancient Near Easterners (Lazaridis et al., 2016), EHG/Steppe ancestry penetrating the region after Bronze Age (Haber et al., 2017, 2020), and African ancestry (represented by Mota; Gallego Llorente et al., 2015) found today in most Levantines and Arabians (Moorjani et al., 2011; Haber et al., 2013). We used seven outgroups in the test: Ust’-Ishim, Kostenki14, WHG, CHG, Natufian (or Levant_N), Papuan, and Mbuti. Standard error range for Levant_N (0.02-0.03); Iran_N (0.02-0.03); EHG (0.01-0.02); Mota (0.005-0.01); Natufian (0.02).

a p > 0.05 indicates the model is not rejected. .HO, samples from the Human Origins dataset. We note here that these ancient populations represent proxies to the actual populations that contributed to the ancestors of modern populations.

In addition to differences in EHG and African ancestries, we observed an excess of Natufian ancestry in Arabia compared with the Levant (Figures 1B and 1E). When we substituted Levant_N with Natufians as source of ancestry in the Middle East, we found that Arabians could be successfully modeled (Table 1), whereas none of the present-day Levantines could be modeled as such. Model-based clustering also showed that Arabian populations have substantially lower Anatolia Neolithic (Anatolia_N) ancestry compared with modern-day Levantines (purple component in Figure 1B). The differences in ancient Anatolian ancestry could be from a limited Levant_N expansion into Arabia, as Levant_N shares significant ancestry with Anatolia_N (Lazaridis et al., 2016), but could also be from post-Bronze Age events with the expansion of populations carrying Anatolia_N ancestry into the Levant (Haber et al., 2020).

**Figure 2:**
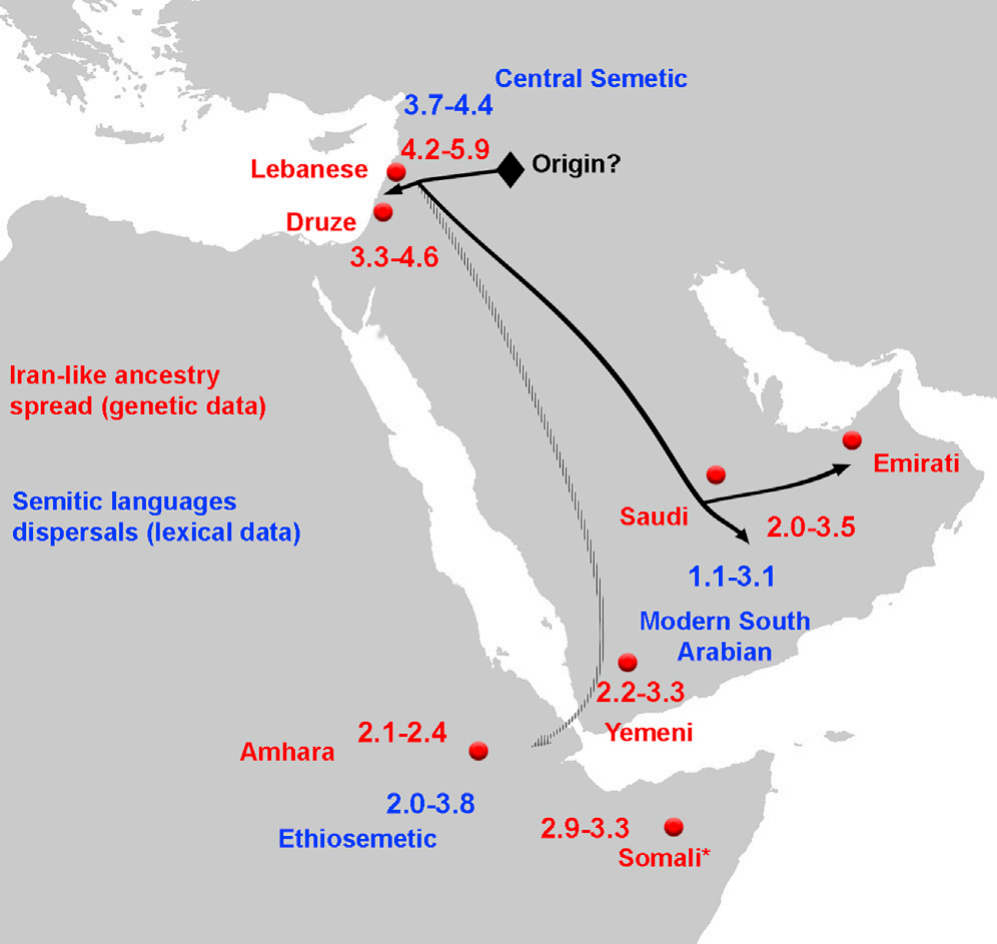
Spread of Iran-like ancestry and Semitic languages Map shows admixture dates in thousands of years ago (red) based on Table S2 and Semitic languages dispersals estimated by Kitchen et al. (2009) from lexical data (blue). Kitchen et al. (2009) estimate an Early Bronze Age origin for Semitic languages ~5.7 KYA in the Levant. Admixture also appears in non-Semitic speaking groups such as the Somalis, a Cushitic-speaking population. Kitchen et al. (2009) suggested that Semitic languages would have spread into East Africa with little gene flow, as Ethiosemitic-speaking populations share similar proportions of non-African ancestry and are genetically similar to Cushitic-speaking populations, confirmed by more recent analysis (Pagani et al., 2015). They proposed that the current distribution of Ethiosemitic languages reflect a language diffusion process through African populations, rather than gene flow. Our admixture tests Tables S3 and S4 also suggest an ancient Egyptian source of ancestry in East Africa, rather than from Arabia, although ancient DNA from Arabia is still missing to make a comparable analysis. See also Figure S2.

**Figure S2:**
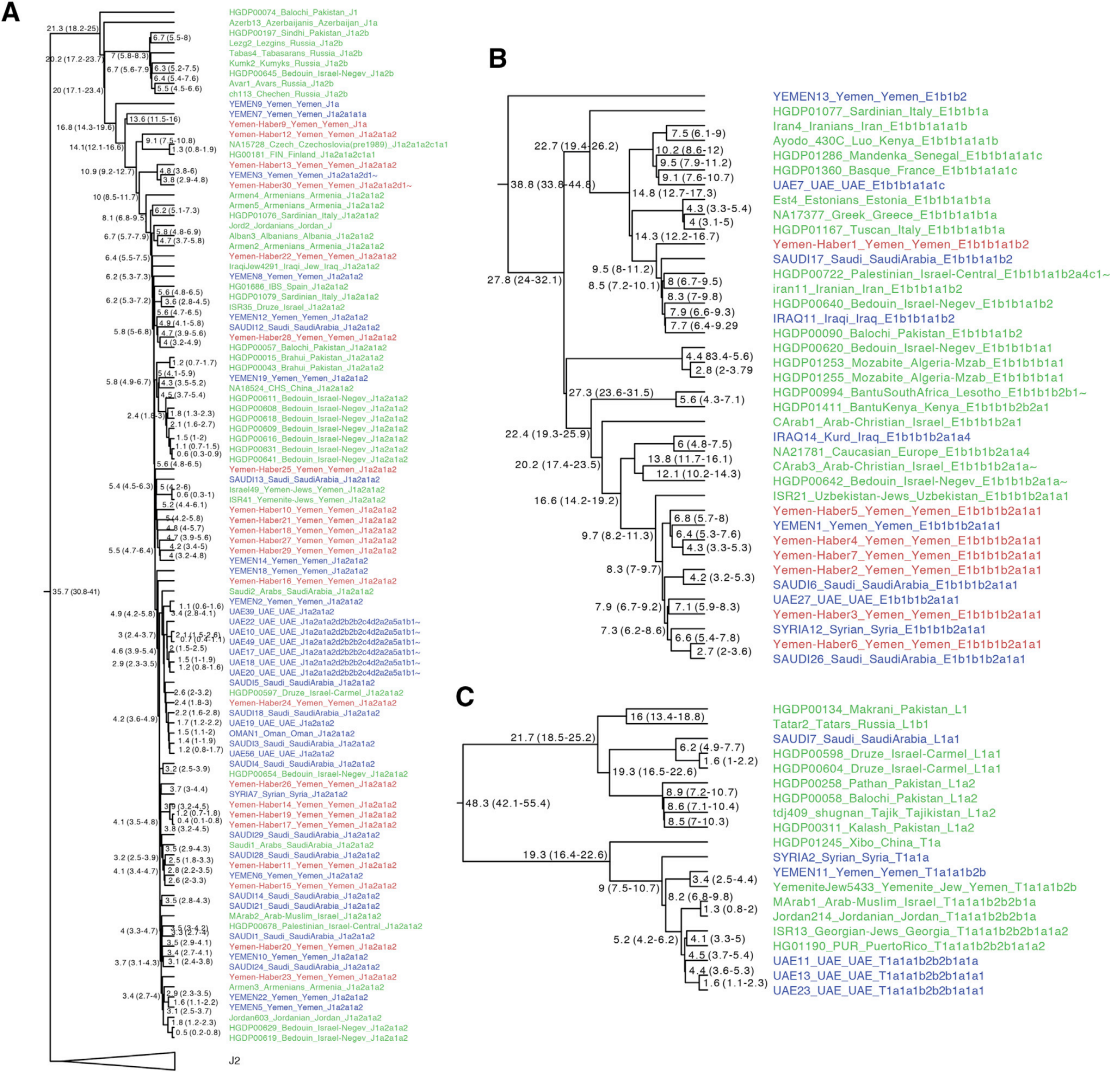
Y chromosome phylogeny, related to Figure 2 We merged our dataset (samples in Blue) with Haber et al., 2019 (samples in Red) and Hallast et al., 2020 (Samples in Green). We display common haplogroups found in our dataset (**A**) J1, (**B**) E1b1 and (**C**) L-T. Numbers at each node represent coalescence date in thousand years with 95% confidence intervals in brackets.

In addition to the local ancestry from Epipaleolithic/Neolithic people, we found an ancestry related to ancient Iranians that is ubiquitous today in all Middle Easterners (orange component in Figure 1B; Table 1). Previous studies showed that this ancestry was not present in the Levant during the Neolithic period but appeared in the Bronze Age where ∼50% of the local ancestry was replaced by a population carrying ancient Iran-related ancestry (Lazaridis et al., 2016). We explored whether this ancestry penetrated both the Levant and Arabia at the same time and found that admixture dates mostly followed a North to South cline, with the oldest admixture occurring in the Levant region between 3,300 and 5,900 ya (Table S2), followed by admixture in Arabia (2,000–3,500 ya) and East Africa (2,100–3,300 ya). These times overlap with the dates for the Bronze Age origin and spread of Semitic languages in the Middle East and East Africa estimated from lexical data (Kitchen et al., 2009; Figure 2). This population potentially introduced the Y chromosome haplogroup J1 into the region (Chiaroni et al., 2010; Lazaridis et al., 2016). The majority of the J1 haplogroup chromosomes in our dataset coalesce around ∼5.6 (95% CI, 4.8–6.5) kya, agreeing with a potential Bronze Age expansion; however, we did find rarer earlier diverged lineages coalescing ∼17 kya (Figure S2). The haplogroup common in Natufians, E1b1b, is also frequent in our dataset, with most lineages coalescing ∼8.3 (7–9.7) kya, though we also found a rare deeply divergent Y chromosome, which coalesces 39 kya (Figure S2).

**Figure 3:**
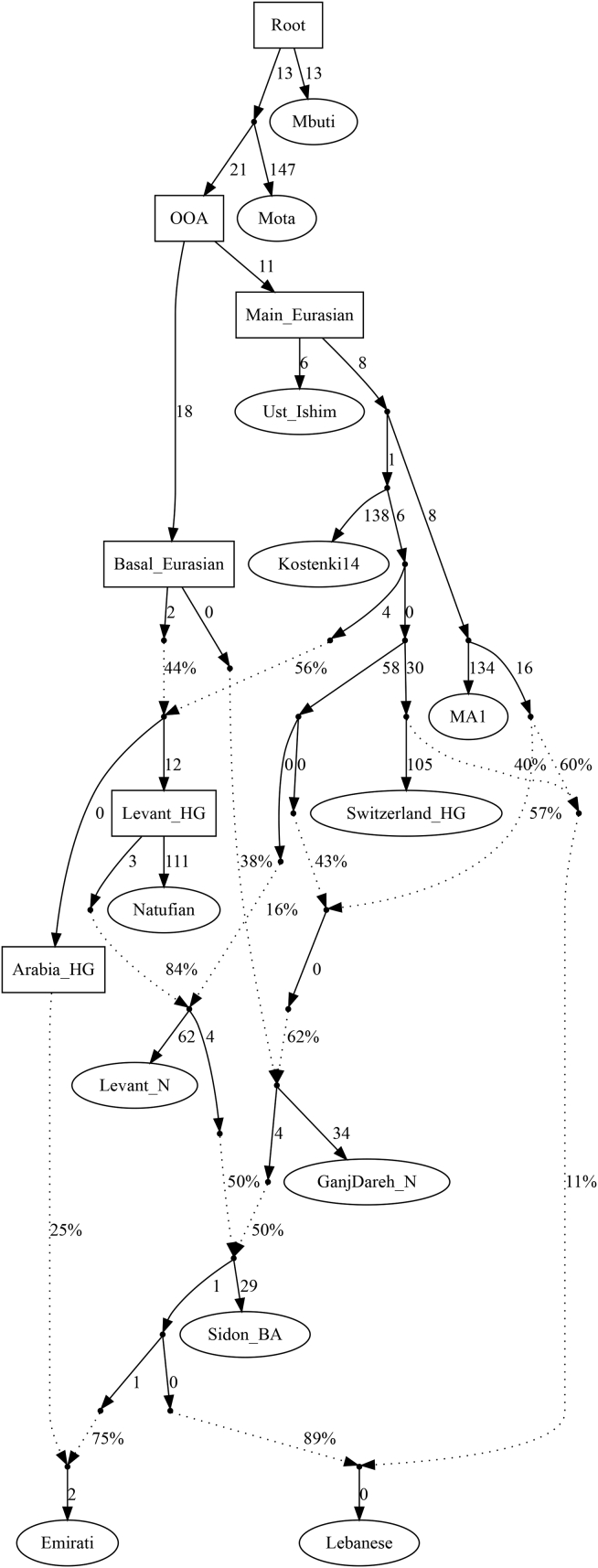
A possible model for the population formation in the Middle East Populations in ellipses are sampled populations, while populations in boxes are hypothetical. Worst f-statistics: *Z* score = −2.9. We explore models further in Figure S3. BA, Bronze Age; HG, hunter-gatherer.

**Figure S3:**
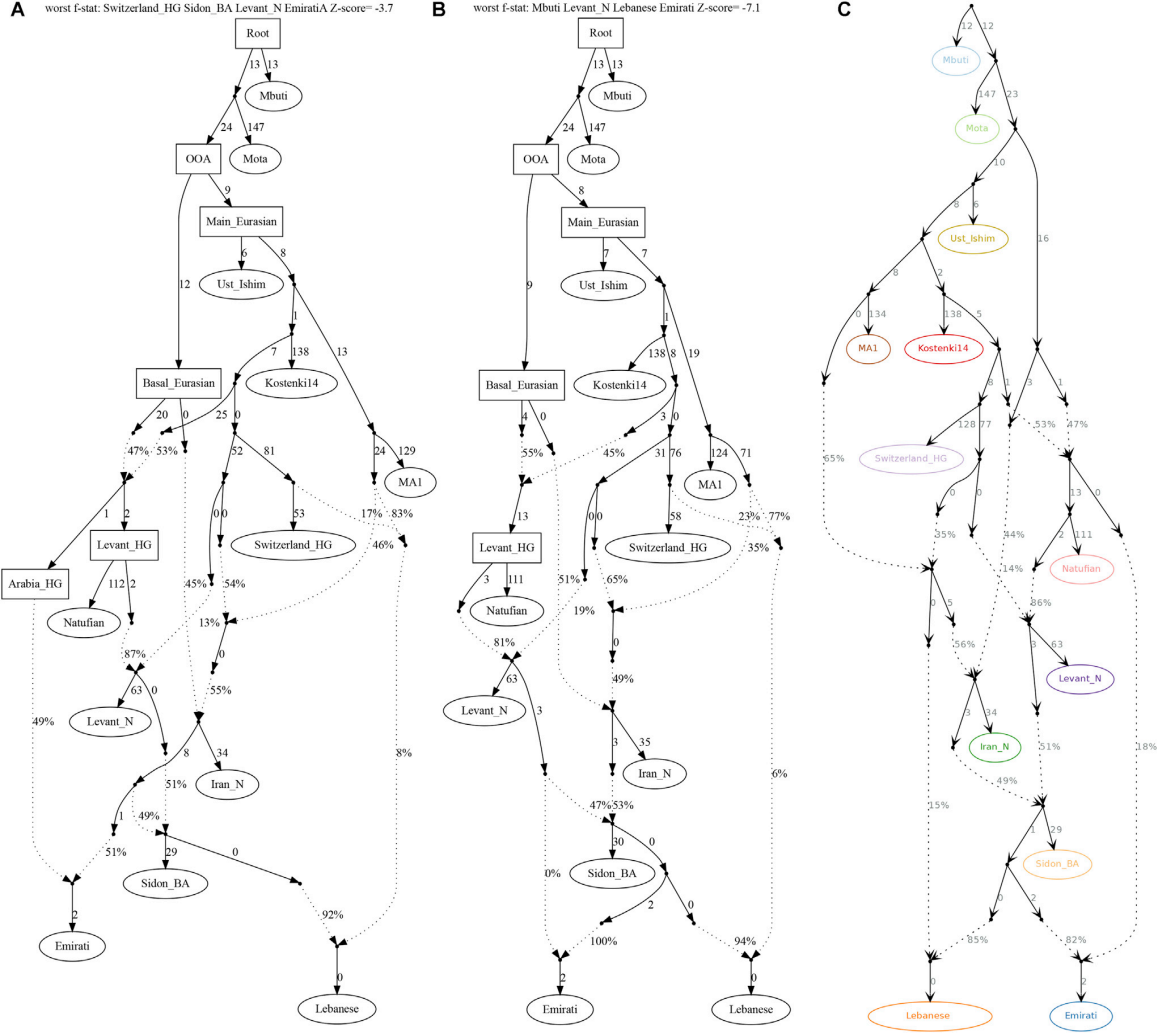
qpGraph alternative models for population formation in the Middle East and automatically fitting admixture graphs, related to Figure 3 Graphs (A) and (B) show alternative scenarios for populating the Middle East. Changes from the best model (Figure 3) involve (A) Arabians derive their ancestry from a population related to ancient Iranians and local hunter-gatherers. (B) Ancestry in Arabia from a Levant_N-related rather than Natufian-related population. (C) We show a semi-automatically fitted graph. We started with a base-graph of the ancient populations based on previous knowledge (Lazaridis et al., 2016; Haber et al., 2017); this graph has an outlier Z-score = 2.06. We then used qpBrute (Ní Leathlobhair et al., 2018; Liu et al., 2019) to fit the EmiratiA and we obtained a graph with no outliers showing EmiratiA descended from a mixture of Natufian-related and Sidon_BA-related ancestries. We then used this new graph as a base and added the modern Lebanese. We found that the graph with the lowest Z-score shown here was identical to our Figure 3.

We next tested whether we can model our populations as deriving ancestry from one of the sampled regional Bronze Age populations and found that the Middle Bronze Age population from Sidon (Sidon_BA) could be a source of ancestry for some modern Levantine and Arabian populations (Tables S3 and S4). Our phylogenetic modeling suggests that modern Levantines could have directly derived their ancestry from a Sidon_BA-related population; however, Arabians require additional ancestry from a Natufian-related population (Figures 3 and S3).

### Effective population size and separation history

**Figure 4:**
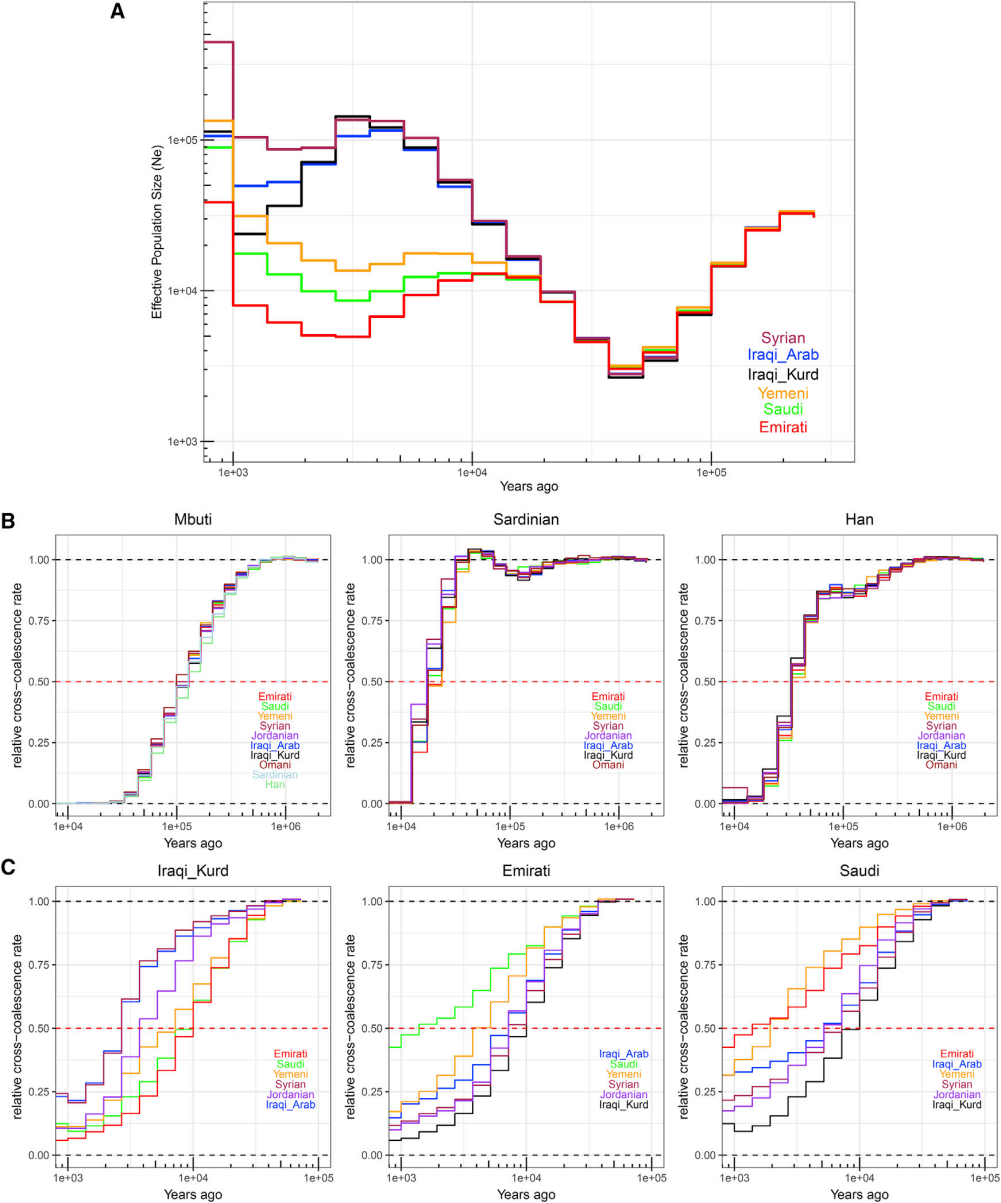
Population size and separation history (A) Effective population size histories for Middle Eastern populations. More details in Figure S4. (B) Separation history between Mbuti, Sardinians, and Han (indicated at the top of each panel) with each of the Middle Eastern populations (identified within each panel). All Middle Eastern populations show similar split time with each of these global populations. More details in Figure S5. (C) Separation history within the Middle East (population indicated at the top of each panel, and within each panel). More comparisons shown in Figure S4. Note the different x axis scales. See also Figure S5.

**Figure S4:**
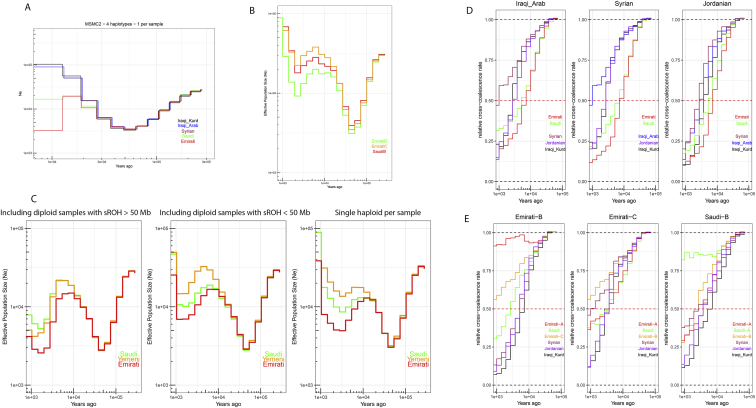
Effective population size and separation history estimates, related to Figure 4 **A)** Replicating the divergence in population size between the Levant and Arabia using MSMC2. (**B**) Effective population sizes for Emirati and Saudi subpopulations using Relate. (**C**): Testing the effect of consanguinity on Emirati-A, Saudi-A and Yemeni population size estimates using Relate. sROH calculated using a minimum ROH block of 1Mb. Including samples with likely recent consanguinity affects populations size estimates at recent times. Using a single haplotype per sample reduces this effect. The second bottleneck is apparent in all tests. (**D** and **E**) Separation history within the Middle East for additional populations. Population indicated at the top of each panel, and within each panel.

Historical effective population sizes can be inferred through the distribution of coalescence times between chromosomes sampled from a population (Li and Durbin, 2011). However, there is limited resolution in recent periods using single human genomes, while errors in haplotype phasing create artifacts when using multiple genomes (Schiffels and Durbin, 2014; Terhorst et al., 2017). Although methods have been developed that extend these approaches by incorporating the allele frequency spectrum from unphased genomes, they do not have resolution at recent times, for example, through the metal ages (Terhorst et al., 2017; Bergström et al., 2020). By leveraging recent advances in generating genome-wide genealogies (Speidel et al., 2019), and the large number of physically phased samples in our study, we could estimate the effective population size of each population in our dataset up to very recent times—1 kya (Figures 4A and S4). We found that the ancestors of all Middle Easterners show a significant decrease in population size around the out-of-Africa event ∼50–70 kya. The recovery from this bottleneck follows a similar pattern until 15–20 kya, when a contrast between the Levant and Arabia started to emerge. All Levantine and Iraqi populations continued to show a substantial population expansion, while Arabians maintained similar sizes. This contrast is noteworthy since it starts after the end of the Last Glacial Maximum and becomes prominent during the Neolithic, when agriculture developed in the Fertile Crescent and led to settled societies supporting larger populations. Following the Neolithic, and with the start of the aridification of Arabia around 6 kya, Arabian populations experienced a bottleneck while Levantines continued to increase in size. The expansion in Levantines then plateaus and their population size decreases around the 4.2 kiloyear aridification event (Weiss et al., 1993). The decline in Emiratis is especially prominent, reaching an effective population size of ∼5,000, more than 20 times smaller than Levantines and Iraqis at the same time period. A recovery can be observed in the past 2 ky. Our results are robust to recent consanguinity common in the region that has likely affected previous population size estimates (Bergström et al., 2020), as we included a single haplotype per sample in our analysis (Figure S4).

**Figure S5:**
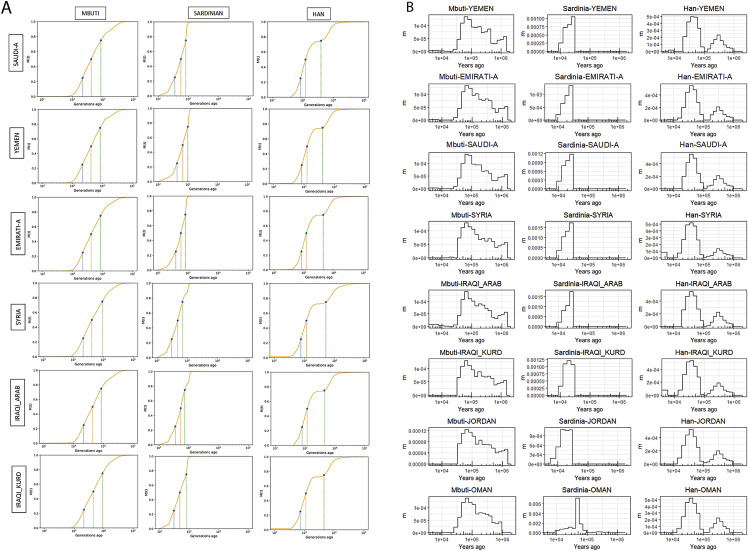
Migration rates inferred using MSMC-IM, related to Figure 4 **A)** Cumulative migration probability, M(t), of Middle Eastern samples compared to Mbuti, Sardinians and Han. Shaded lines illustrate when the M(t) reaches, 25%, 50% and 75%. (**B**) Migration rates, m, for the same populations. Note the gradual separation from Mbuti, more of a clean split from Sardinians and the second, older, peak found in the Han comparisons which are consistent with archaic hominin lineages.

We next studied the population separation history of Middle Eastern populations among themselves and from global populations. The importance of accurate phasing in this analysis is illustrated by an earlier finding that suggested, based on statistically phased data, that modern-day Papuans harbor ancestry of an early expansion of modern humans out of Africa (Pagani et al., 2016). However, this was not replicated using physically phased genomes, suggesting it was caused by a statistical phasing artifact (Bergström et al., 2020). Conversely, when exploring population separation history at recent times, rare variants become more informative but are less accurately phased by statistical methods and are unlikely to be present in reference panels. We first tested whether present-day Middle Easterners harbor ancestry from an early human expansion out of Africa by comparing the split times of our populations with physically phased samples from the HGDP (Figures 4B and S5). Using a relative cross-coalescent rate (rCCR) of 0.5 as a heuristic estimate of split time, we found that Levantines, Arabians, Sardinians, and Han Chinese share the same split time, and additionally the same gradual pattern of separation, from Mbuti ∼120 kya. We then compared the populations in our dataset with Sardinians and found they split ∼20 kya, with Levantines showing a slightly more recent divergence than Arabians. In contrast to the gradual separation patterns to Mbuti, Sardinians show more of a clean split to all Middle Eastern populations. Notably, all lineages within the Levant and Arabia, and in addition to lineages within all Middle Eastern populations and Sardinians, coalesce within 40 kya. These results collectively suggest that present-day Middle Eastern populations do not harbor any significant traces from an earlier expansion out of Africa, and all descend from the same population that expanded out of the continent ∼50–60 kya.

We then compared the separation times of populations within the Middle East and found the oldest divergence times were between Arabia and the Levant/Iraq (Figures 4C and S4). The Emiratis split from Iraqi-Kurds around 10 kya, and more recently around 7 kya from Jordanians, Syrians, and Iraqi-Arabs. Saudi split times from the same populations appear more recent, around 5–7 kya, while the Yemeni separation curves are intermediate between the Emirati and Saudi curves. The split times between Arabia and the Levant predate the Bronze Age, agreeing with our phylogenetic modeling that, if a Bronze Age expansion into Arabia occurred, it did not result in a complete replacement of ancestry.

Within the Levant and Iraq, all splits occurred in the past 3–4 ky. Within Arabia, Yemenis split from Emiratis ∼4 kya and Saudis appear as the least divergent population to both the Emiratis and Yemenis, with recent splits within the last 2 ky. We note that the separation curves within the region appear gradual, suggesting ongoing gene flow after separation rather than clean splits. We also note that the separation curves will reflect the admixture histories of these populations.

### Archaic introgression and deep ancestry in the Middle East

**Figure 5:**
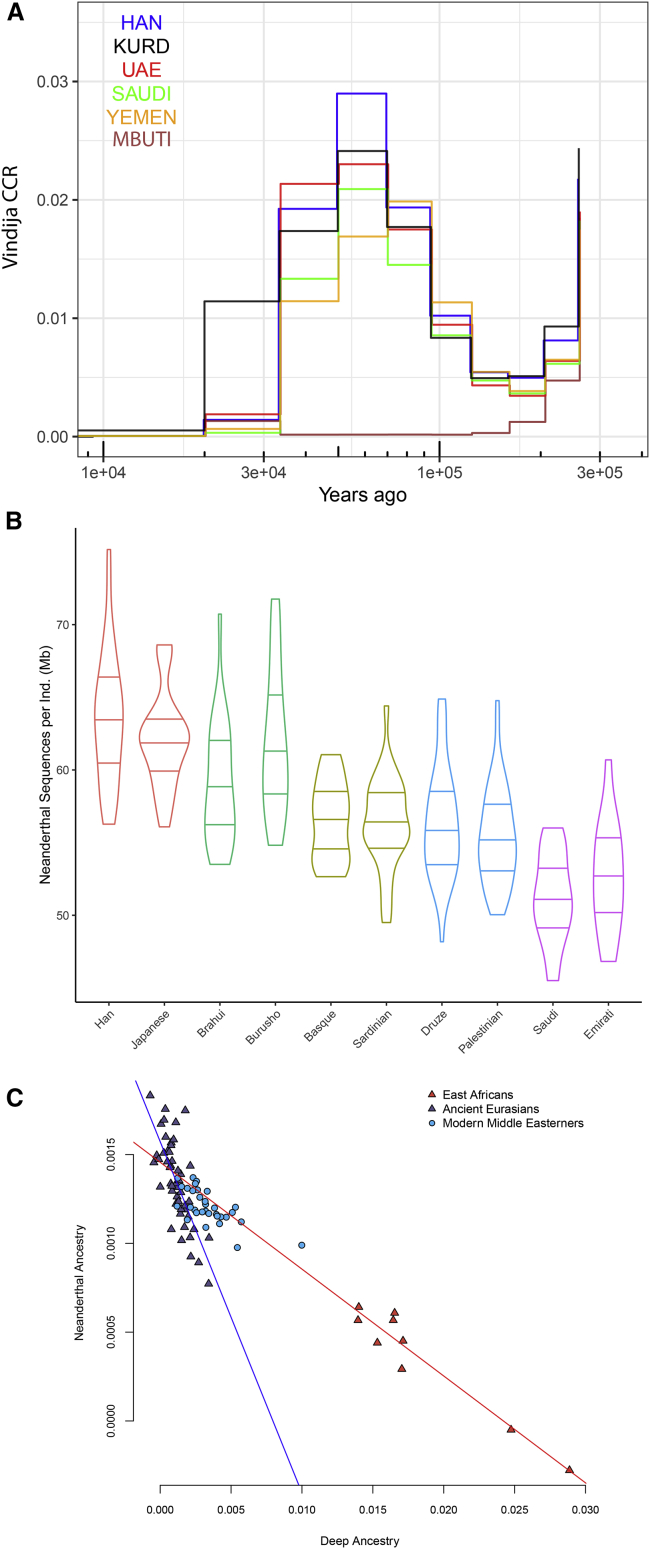
Archaic introgression and deep structure in the Middle East (A) Relative cross coalescent rate (CCR) against Vindija Neanderthal. Note the y axis range. (B) Distribution of total length of Neanderthal sequences (Mb) per sample in each population. Horizontal lines depict 25%, 50%, and 75% quantiles. Colors reflect regional grouping. (C) Neanderthal ancestry *f4*(Vindija,Chimp;X,Mbuti) is negatively correlated with a deep ancestry *f4*(Kostenki14,X;Ust'-Ishim,Chimp) in the Middle East. Two clines explain the depletion of Neanderthal Ancestry in Middle Easterners; one formed by Basal Eurasian ancestry and the other is African ancestry. We plot regression lines using East Africans (red) and the ancient Eurasians (blue). We generated standard errors for the slopes using a jackknife by dropping one chromosome. Ancient Eurasian slope m = −0.21 ± 0.002, East African slope m = −0.06 ± 0.0008. Both slopes are always negative. See also Figure S6.

The similar amount of Neanderthal ancestry in most non-African populations and the low diversity of introgressed haplotypes suggest that modern humans likely experienced a single pulse of Neanderthal admixture as they expanded out of Africa (Bergström et al., 2020). Middle Eastern populations have previously been shown to have lower Neanderthal ancestry than European and East Asian populations (Rodriguez-Flores et al., 2016; Bergström et al., 2020); however, the interpretation of this finding is complicated by recent African admixture “diluting” Neanderthal ancestry (Haber et al., 2016). In addition, some analyses require the use of an outgroup, which, if itself contains Neanderthal ancestry, can bias estimates (Chen et al., 2020). To investigate Neanderthal introgression in our dataset, we exploited the accurate phasing of our samples and compared cross-coalescent rates with the high coverage Vindija Neanderthal genome (Prüfer et al., 2017). All Middle Easterners showed an archaic admixture signal at a time point similar to other Eurasians (Figure 5A).

**Figure S6:**
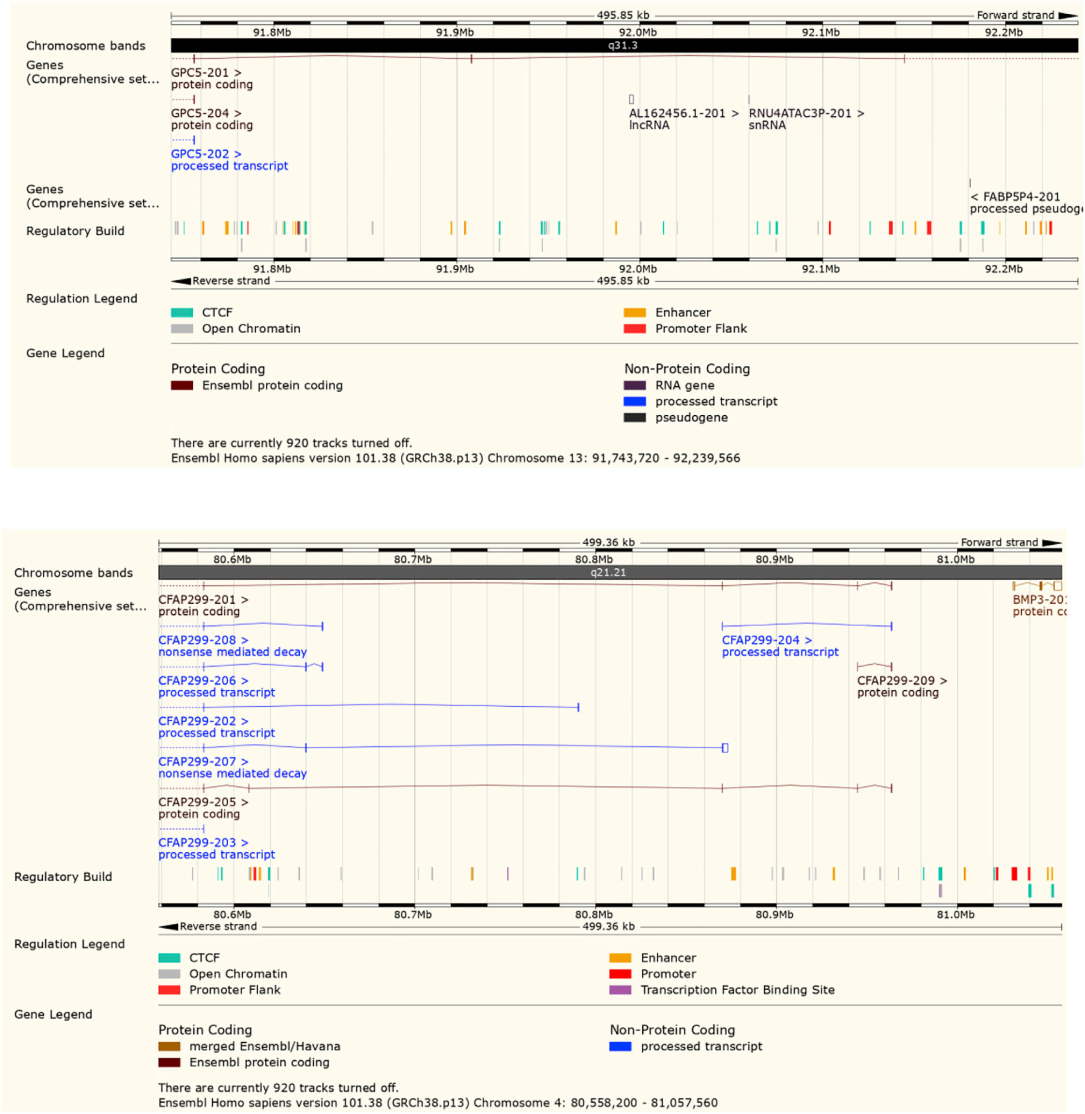
Neanderthal introgressed segments common in Arabia but rare globally identified using Sprime, related to Figures 5A and 5B Top: 496kb segment on chromosome 13 present at ~20% frequency in Saudi populations but rare globally (Global 1000G Project = 0.02%) and overlapping *GPC5*, a gene expressed in brain tissues. Bottom: 499kb segment on chromosome 4 that reaches ~20% frequency in EmiratiA and overlaps *CFAP299* expressed in the testes with a role in spermatogenesis, and *BMP3*, a cytokine which induces cartilage and bone development (Global 1000G Project < 0.05%). We searched for functional variants within these haplotypes but did not find any amino acid changes within canonical transcripts, with most substitutions limited to introns. Figures downloaded from Ensembl.

We then used an identity-by-descent-based method, IBDmix, which directly compares a target population to the Neanderthal genome to detect haplotypes of Neanderthal origin (Chen et al., 2020). We ran IBDmix on our samples and the HGDP dataset, recovering segments totaling ∼1.27 Gb that are of likely Neanderthal origin. When comparing the amount of Neanderthal haplotypes that are private to our dataset but not present in other non-Middle Eastern Eurasians, we found only ∼25 Mb in total, illustrating that the vast majority of Neanderthal haplotypes in the region are shared with other populations. However, we did find relatively large introgressed haplotypes (∼500 kb) that are very rare globally but reach high frequencies in Arabia (Figure S6). We then compared the average number of total Neanderthal bases per population and found lower values in Arabia in comparison to other Eurasian populations, including Levantines. The Druze and Sardinians, for example, have similar amounts (average ∼56.4 Mb per individual) of Neanderthal ancestry (Figure 5B). In contrast, in Arabia, EmiratiA and SaudiA have an average of 52.7 and 52.1 Mb Neanderthal ancestry, respectively, which is ∼8% lower than the Druze and Sardinians, and ∼20% less than Han Chinese. Since EmiratiA and SaudiA have less than 3% of African ancestry (Table 1), the depletion of Neanderthal ancestry in Arabia cannot be explained by the African ancestry alone. Lazaridis et al., (2016) proposed that a basal Eurasian population, with low-to-no Neanderthal ancestry, had contributed different proportions to ancient and modern Eurasians, reaching ∼50% in Neolithic Iranians and Natufians. Since Arabians have an excess of Natufian-like ancestry compared to elsewhere in the Middle East, we found they also carry an excess of basal Eurasian ancestry that will reduce their Neanderthal ancestry. In addition, most modern Middle Easterners carry African ancestry from recent admixture, which also contributes to their deep ancestry (relative to the time of a main Eurasian ancestry). We found a negative correlation (Pearson’s r = −0.7, p = 9.1 × 10^−6^) between the increase in deep ancestry and the amount of Neanderthal ancestry in the modern Middle Easterners. When testing all ancient populations, we found two clines (Figure 5C) explaining the depletion of Neanderthal ancestry: the first is formed by African ancestry while the second is formed by a Basal Eurasian ancestry in ancient Eurasians. Middle Easterners appear to be affected by both clines since they harbor both ancestries.

### Selection

**Figure 6:**
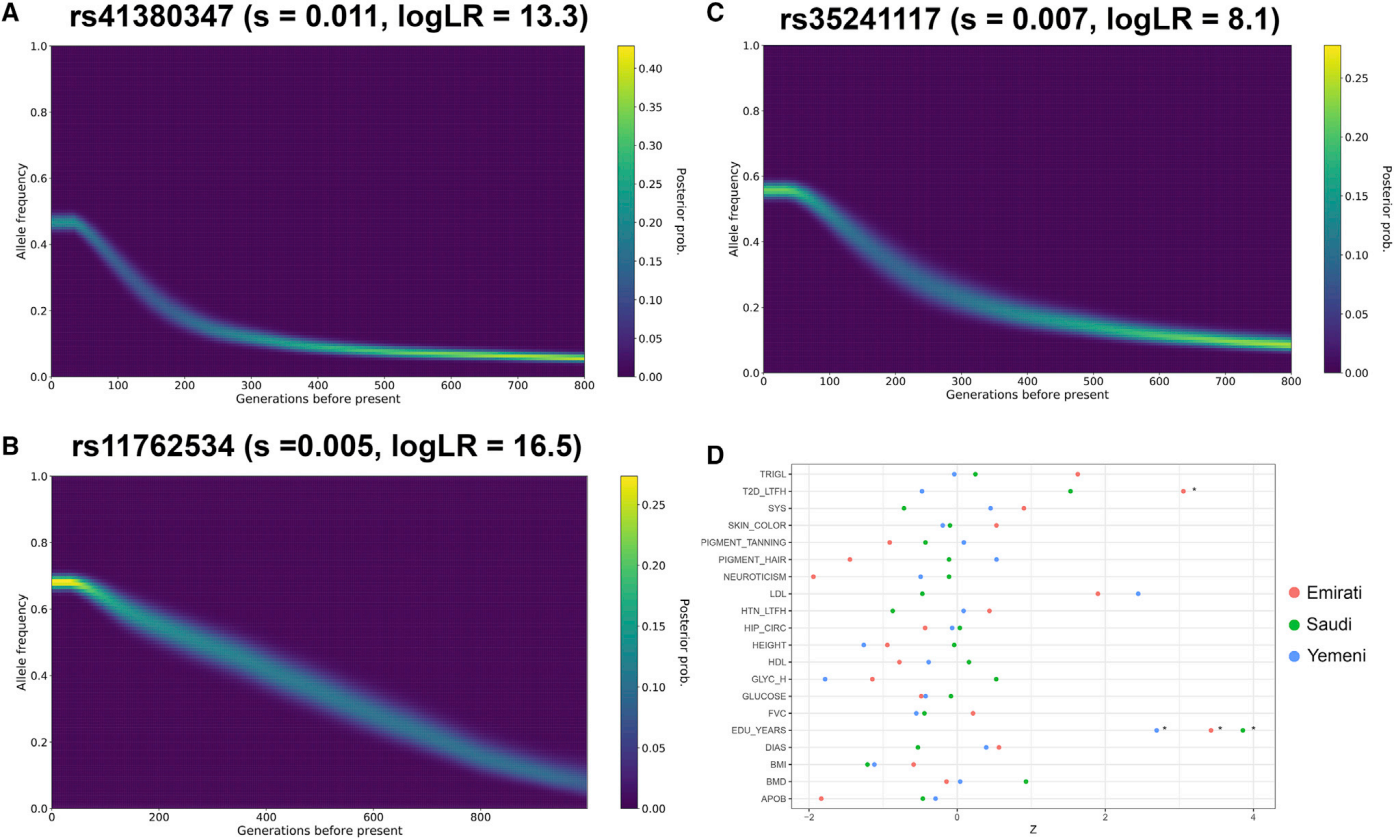
Selection in Arabia (A) Historical allele trajectory of rs41380347, which is associated with lactase persistence and almost private to the Middle East. s, selection coefficient. (B) Frequency trajectory of rs11762534, which is associated with lymphocyte and neutrophil percentages and prostate neoplasm malignancy. (C) Frequency trajectory of rs35241117, which is present at the highest frequency in Arabia globally and is associated with multiple traits including glomerular filtration rate, bone mineral density, BMI, standing height, and hypertension. (D) Testing for recent polygenic selection, over the past 2,000 years, on 20 traits within Arabian populations. Asterisks indicate the test is significant after correcting for multiple testing (FDR = 5%). TRIGL, triglycerides; T2D, type 2 diabetes; SYS, systemic blood pressure; LDL, low-density lipoproteins; HTN, hypertension; HIP_CIRC, hip circumference; HDL, high-density lipoproteins; GLYC_H, glycosylated *haemoglobin*; FVC, forced vital capacity; EDU_YEARS, years of education; DIAS, diastolic blood pressure; BMI, body mass index; BMD, bone mass density; APOB, Apoliprotein B. See also Figure S7.

The current hyper-arid climate may have potentially exerted selective pressure for adaptations in Arabian populations. To explore this, we searched genome-wide genealogies for lineages carrying mutations that have spread unusually quickly (Speidel et al., 2019) at a conservative genome-wide threshold (p < 5 × 10^−8^). Previous studies identified two correlated variants (rs41380347 and rs55660827), distinct from the known European variant (rs4988235), that are associated with lactase persistence in Arabia (Imtiaz et al., 2007; Enattah et al., 2008). For the Arabian variant rs41380347, we found evidence for strong selection (Figure 6A, s = 0.011, logLR = 13.3), similar to, but slightly weaker than, the reported strength of selection at rs4988235 in Europeans (s = 0.016–0.018; Mathieson and Mathieson 2018; Stern et al., 2019). The variant is present at highest frequency in the Arabian populations: ∼50% in Saudis and Emiratis, and at a much lower frequency in the Levant and Iraq (4%). Remarkably, the variant is not present in any Eurasian or African population in the 1000 Genomes Project (1KG), although it is found at low frequency in some East African groups (Tishkoff et al., 2007). We also did not find the variant in 157 published ancient Eurasian whole genomes (Broushaki et al., 2016, de Barros Damgaard et al., 2018, Haber et al., 2017, Haber et al., 2019a, Haber et al., 2020, Jones et al., 2015), including ancient Levantines and Iranians, consistent with a recent origin of the haplotype within the Middle East and subsequent increase in frequency due to selection. We found the variant had a rapid increase in frequency between 9 kya and the present day (Figure 6A). Notably, this period overlaps with the transition from a hunter-gatherer to a herder-gatherer lifestyle in Arabia (Petraglia et al., 2020).

We also identified additional variants that show an increase in frequency recently. A variant within *LMTK2*, rs11762534, which is also an eQTL for many genes, displays evidence of putative selection (Figure 6B, s = 0.005; logLR = 16.5). *LMTK2* encodes a serine/threonine kinase that is implicated in diverse cellular processes including apoptosis, growth factor signaling and appears essential for spermatogenesis in mice Cruz et al., 2019, Kawa et al., 2006. Outside the Middle East the variant is highly stratified and is present at the highest frequency in Europeans (1KG, 45%), while rare in Africans and East Asians (<1%). We found it at 66% frequency in the Arabian populations and in BedouinB (81%), while appearing less frequent in Druze and Palestinians (both ∼55%). We also found a signal of strong putative selection at rs35241117 (Figure 6C, s = 0.007, logLR = 8.1). This variant shows the highest global frequency in Saudis and Yemenis (∼60%) and is associated with a number of metabolic, skeletal, and immunological traits, including glomerular filtration rate, diuretics, hypertension, and BMI (Watanabe et al., 2019; Canela-Xandri et al., 2018). rs35241117 lies outside a ∼400 kb haplotype that has recently been suggested to be under selection in Kuwaitis and Saudis (Eaaswarkhanth et al., 2020) but is in moderate LD (r^2^ = 0.51) with it.

**Figure S7:**
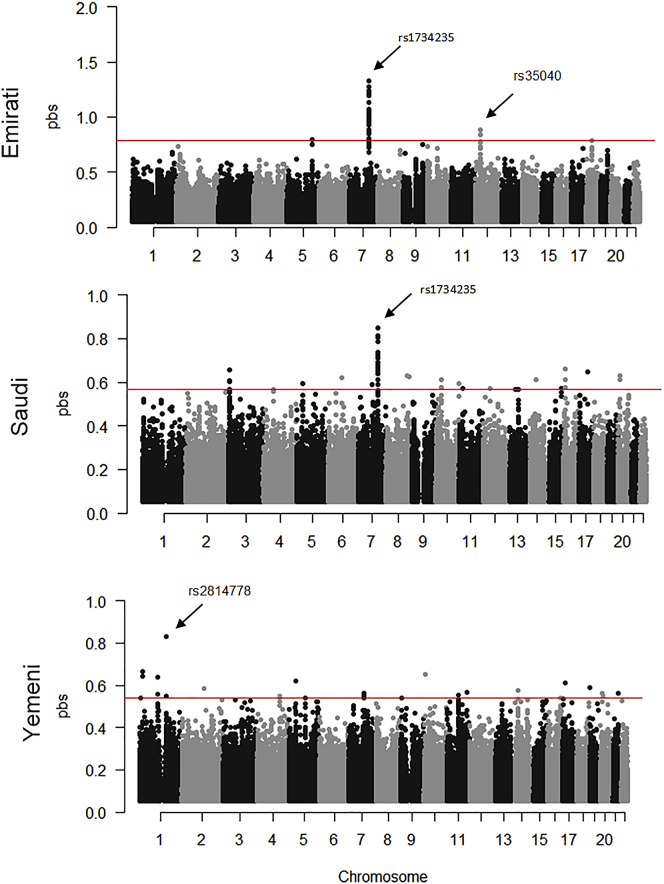
Population Branch Statistics comparing each Arabian (EmiratiA, SaudiA, Yemeni) population with Iraqi_Arabs and using Syrians as an outgroup, related to Figure 6 Variants showing extreme branch statistics highlighted. Red line illustrates the top 99.999% quantile. Note the different y axis scales. rs2814778 is the variant discussed in the main text found at high frequencies in Yemenis that results in the Duffy null genotype. rs35040 shows strong differentiation in Emiratis and is an eQTL for *DDX11* in multiple tissues. For both Emiratis and Saudis, we find a strong signal of differentiation at a 97kb haplotype on chromosome 7. Variants on this haplotype (rs1734235) almost reach fixation (97% and 85%, in Emiratis and Saudis respectively) and are associated with increased expression of the lincRNA AC003088.1 in cultured fibroblasts (GTEx Analysis Release V8; The GTEx Consortium, 2020).

We additionally looked for strongly differentiated variants between Arabia and the Levant/Iraq (Figure S7). For both Emiratis and Saudis, we found a strong signal of differentiation at a 97kb haplotype on chromosome 7 (Figure S7). Variants on this haplotype (rs1734235) almost reach fixation in Arabians and are associated with increased expression of the lincRNA AC003088.1 in cultured fibroblasts (GTEx Analysis Release V8; The GTEx Consortium, 2020). The most extreme population branch statistic in Yemenis is rs2814778 (Figure S7), where the derived allele results in the Duffy null phenotype and is almost exclusively found in African populations in the 1000 Genomes Project. However, the variant is very common in Yemenis (74%), and decreases in frequency moving northward in the peninsula (59% in Saudis while reaching 6% in Iraqi-Arabs). We found that across the genome this region shows the highest enrichment of African ancestry in the Middle East, in agreement with a previous study (Fernandes et al., 2019). As the average amount of African ancestry in Yemenis and Saudis is ∼9% and ∼3% respectively (Table 1), the high frequency of this variant appears consistent with positive selection after African admixture. It has been thought that the derived allele protects against *Plasmodium vivax* infection (Miller et al., 1976), which has been historically present in Arabia.

An advantage of using genome-wide genealogies is its power to detect relatively weak selection. We subsequently searched for evidence of polygenic adaptation in Arabian populations across 20 polygenic traits specifically over the past 2,000 years (STAR Methods). For most traits, we found no, or inconclusive, evidence for recent directional selection, including height, skin color, and BMI (Figure 6D). However, a few traits do show evidence, with the strongest putative selection signal appearing on genetic variants associated with higher years of education in present-day Western societies (EduYears) consistent across all Arabian populations (p = 0.0002 in Saudis). This has also been reported in the British population (Stern et al., 2021); however, the signal was shown to become attenuated after conditioning on other traits, suggesting indirect selection via a correlated trait. In contrast to findings in the British population (Stern et al., 2021), we do not find evidence of putative selection acting on traits such as sunburn, hair color, and tanning ability. Within Arabia, the direction of putative selection on most traits appears to be similar across populations, likely as a result of shared ancestry; however, we note that the current varied environments across the region can potentially cause different recent selective pressures. In Emiratis, we found evidence of putative selection on variants increasing type 2 diabetes (T2D, p = 0.004). This result is intriguing, as the prevalence of T2D in Emiratis is among the highest globally and is partly thought to result from strong recent shift to a sedentary lifestyle (Malik et al., 2005). We also found nominal evidence of putative selection acting to increase levels of low-density lipoproteins (LDL; p = 0.01) and decrease levels of Apoliprotein B (APOB; p = 0.01) in the same population, but, they appear suggestive after adjusting for multiple testing (*P*_*adj*_ = 0.06 at 5% FDR).

## Discussion

In this study, we have generated a high-coverage resource from the genetically understudied Middle East region. All samples studied are experimentally phased using linked-read sequencing, allowing the reconstruction of large and accurate haplotypes. We found millions of variants that are not cataloged in previous global sequencing projects, with a significant proportion being common in the Middle East. A majority of these common variants reside outside of short-read accessibility masks, highlighting the limitation of standard short-read sequencing based studies.

The large number of physically phased haplotypes allowed us to study population history from relatively old periods (>100 kya) to very recent times (1 kya). We found no evidence that an early expansion of humans out of Africa has contributed genetically to present-day populations in the region. This finding adds to the growing consensus that all contemporary non-African modern humans descend from a single expansion out-of-Africa, quickly followed by admixture with Neanderthals, before populating the rest of the world (Mallick et al., 2016; Bergström et al., 2020). We found that Middle Eastern populations have very little Neanderthal DNA that is private to the region, with the vast majority shared with other Eurasians. We demonstrated that Arabian populations have lower Neanderthal ancestry than Levantine, European, and East Asian populations and attributed this difference to elevated ancestry from a basal Eurasian population, which did not admix with Neanderthals, in addition to recent African admixture.

By modeling contemporary populations using ancient genomes, we identified differences between the Levant and Arabia. The Levant today has higher European/Anatolian-related ancestry while Arabia has higher African and Natufian-like ancestries. The contrast between the regions is also illustrated by their population-size histories that diverged before the Neolithic (15–20 kya) and suggest that the transition to a sedentary agricultural lifestyle allowed the growth of populations in the Levant but was not paralleled in Arabia. It has been suggested that population discontinuity occurred between the late Pleistocene and Early Holocene in Arabia and that the peninsula was repopulated by Neolithic farmers from the Fertile Crescent (Uerpmann et al., 2010). Our results do not support a complete replacement of the Arabian populations by Levantine farmers. In addition, our models suggest that Arabians could have derived their ancestry from Natufian-like local hunter-gatherer populations instead of Levantine Farmers. The identification of lithic assemblages in Northern Arabia, some of which appear similar to ones made by Levantine farmers (Crassard and Drechsler, 2013a), in addition to the movement of animal domesticates between the Levant and Arabia, have been suggested to occur either due to population movements or through cultural diffusion (Guagnin et al., 2017; Petraglia et al., 2020). Our results suggest the latter scenario and/or limited migration from the Levant.

An additional source of ancestry needed to model modern Middle Easterners is related to ancient Iranians. Our admixture tests show that this ancestry first reached the Levant and subsequently reached Arabia and East Africa. The timings of these events interestingly overlap with the origin and spread of the Semitic languages (Kitchen et al., 2009), suggesting a potential population carrying this ancestry (possibly unsampled yet from the Levant or Mesopotamia) may have spread the language. We found climate change associated aridification events to coincide with population bottlenecks, with Arabians decreasing in size ∼6 kya with the onset of the desert climate while Levantines around the 4.2 kiloyear aridification event. This severe drought has been suggested to have caused the collapse of kingdoms and empires in the Middle East and South Asia, potentially reflected genetically in the signal we identify (Weiss, 2017).

The application of ancestral recombination graphs to reconstruct the evolutionary history of variants offers a powerful method to study natural selection. We refined and identified signals of selection in Arabian populations. The example of the lactase persistence associated variant, which during the past few thousand years increased to a frequency reaching 50% and is almost absent outside the region, demonstrates the importance of studying underrepresented populations to understand human history and adaptation. Our results indicate that polygenic selection might have played a role in increasing the frequency of variants that were potentially beneficial in the past but today are associated with diseases such as T2D. We found few signals of polygenic selection in Arabian populations relative to other populations (Speidel et al., 2019; Stern et al., 2021), which may be a consequence of their long-term small effective population sizes that will theoretically reduce the strength of selection (Speidel et al., 2019). The long-term small effective population size, especially coupled with the recent practice of consanguinity, can be exploited for the study of Mendelian and complex traits, as individuals are more likely to carry homozygous loss-of-function mutations and serve as natural “human knockouts.” Our study and the recent establishment of national biobanks in the region are a step forward to reduce health disparities and offer an exciting opportunity to explore, in the future, complex, and disease traits in the Middle East.

### Limitations of the study

Future ancient DNA studies from Arabia are needed to refine the formation of Arabian populations and to further clarify prehistorical connections between the Levant and Arabia. As Middle Eastern populations are among the most underrepresented in GWAS (Sirugo et al., 2019), this limits the understanding of selection signals and the analysis of polygenic traits. Future GWAS on Middle Eastern groups are needed to understand the effects of polygenic selection in these populations.

## STAR★Methods

### Key resources table

**Table:** 

Reagent or resource	Source	Identifier
**Critical commercial assays**		

Oragene DNA - OG-600	DNA Genotek	OG-600
MagAttract HMW kit	QIAGEN	Cat No. 67563
Chromium Genome Reagent Kit	10X Genomics	N/A

**Deposited data**		

HGDP SNV Callset	Bergström et al., 2020	ftp://ngs.sanger.ac.uk/production/hgdp/hgdp_wgs.20190516/
Allen Ancient DNA Resource Callset	David Reich Lab	https://reich.hms.harvard.edu/allen-ancient-dna-resource-aadr-downloadable-genotypes-present-day-and-ancient-dna-data
Lebanese population sequencing data	Haber et al., 2017	EGAS00001002084
Ethiopian populations sequencing data	Pagani et al., 2015	EGAS00001000482, EGAS00001000238, EGAS00001000237
Middle Eastern sequencing populations data	This study	ENA:ERP110713

**Software and algorithms**		

Long Ranger pipeline v2.2.2 (GATK v3.7)	10x Genomics	https://support.10xgenomics.com/genome-exome/software/downloads/latest
GraphTyper v2.0	Eggertsson et al., 2017	https://github.com/DecodeGenetics/graphtyper
plink v1.9	Chang et al., 2015	https://www.cog-genomics.org/plink/
covstats	Pedersen et al., 2017	https://github.com/brentp/goleft/
bcftools v1.9	N/A	https://samtools.github.io/bcftools/
CrossMap v0.4.2	Zhao et al., 2014	https://crossmap.readthedocs.io/en/latest/
BEAST v1.8.4	Drummond and Rambaut 2007	https://beast.community/2016-06-17_BEAST_v1.8.4_released.html
RAxML v8.2.10	Stamatakis 2014	https://cme.h-its.org/exelixis/web/software/raxml/
FigTree v1.4.4	N/A	http://tree.bio.ed.ac.uk/software/figtree/
Chromopainter/FineSTRUCTURE pipeline v4.1.1	Lawson et al., 2012	http://paintmychromosomes.com/
(fast)GLOBETROTTER	Hellenthal et al., 2014	http://paintmychromosomes.com/
MALDER v1.0	Loh et al., 2013; Pickrell et al., 2014	https://github.com/joepickrell/malder/tree/master/MALDER
smartpca v16000 (EIGENSOFT)	Patterson et al., 2006	https://github.com/DReichLab/EIG
DyStruct v1.1.0	Joseph and Pe’er, 2019	https://github.com/tyjo/dystruct
qpDstat v755, qpAdm v810, and qpGraph v6450 (ADMIXTOOLS)	Patterson et al., 2012	https://github.com/DReichLab/AdmixTools
RELATE v1.1	Speidel et al., 2019	https://myersgroup.github.io/relate/
MSMC2 v2.1.1	Schiffels and Durbin, 2014; Wang et al., 2020	https://github.com/stschiff/msmc2
MSMC-IM	Wang et al., 2020	https://github.com/wangke16/MSMC-IM
IBDMix	Chen et al., 2020	https://github.com/PrincetonUniversity/IBDmix
Sprime	Browning et al., 2018	https://github.com/browning-lab/sprime
Eaglev2.4.1	Loh et al., 2016	https://alkesgroup.broadinstitute.org/Eagle/downloads/
BEAGLEv4.0	Browning and Browning, 2007	https://faculty.washington.edu/browning/beagle/b4_0.html
CLUES	Stern et al., 2019	https://github.com/35ajstern/clues
PALM	Stern et al., 2021	https://github.com/35ajstern/palm
RFMix v2.03	Maples et al., 2013	https://github.com/slowkoni/rfmix

### Resource availability

#### Lead contact

Further information and requests should be directed to Mohamed A. Almarri (ma17@sanger.ac.uk).

#### Materials availability

This study did not generate new reagents.

### Experimental model and subject details

This study was approved by Dubai Scientific Research Ethics Committee (DSREC-SR-02/2018_01) and by the Wellcome Sanger Institute Human Materials and Data Management Committee (HMDMC 18/026). All individuals who donated samples for this project were interviewed and provided consent for participation and were recruited in the UAE. We use the term ‘Arabian’ in this study to refer to samples from the Arabian Peninsula (Emirati, Saudi and Yemen), Levantine for Syrians and Jordanians, and Iraqi-Arabs and Iraqi-Kurds for samples from Iraq. Saliva samples were collected using the Oragene DNA kits (OG-600) and DNA was subsequently extracted using the QIAGEN MagAttract HMW kit (Cat No. 67563). Fragment sizes and quality were determined through a pulsed-field capillary electrophoresis (Femto Pulse system). Libraries were prepared using 10X Genomics Chromium kits and each sample was sequenced in a separate lane on a HiSeq X instrument.

### Method details

#### Samples processing and quality control

We ran the Long Ranger pipeline (version 2.2.2, using GATK v3.7) to process raw fastq files into phased BAM files and phased VCF files. The average sequencing depth for all samples was 32x, median 31x, calculated using covstats (https://github.com/brentp/goleft/) on the phased BAMs. For each VCF we assessed the number of phased variants through summary.csv file output by Long Ranger, we found on average ∼98% of variants to be physically-phased in each sample. For each VCF we assessed the quality of variant calls output by Long Ranger using the QUALITY filter for each variant. The pipeline uses the haplotype structure informed by the physical phasing to tag variants that are likely to be false positives, as each haplotype can only have one allele. We find that variants that do not have a PASS quality label tend to have low Ts/Tv values, suggesting they contain false positives. We used a stringent filter by setting all non-PASS variants to missing and merged all samples using bcftools v1.9 merge option −0. We removed variants that show excessive heterozygosity as calculated by bcftools ExcHet tag (< 1e-6) and then set any variant with genotype quality (GQ) < 20 and variants located in regions more than twice the average sample depth to missing. The final dataset composed of 23.1 million single nucleotide variants (SNVs). The Ts/Tv after quality control was 1.97, and remained consistent throughout different allele frequency bins suggesting that the variants are of high quality. The TS/TV for all newly identified variants outside the accessibility mask is 1.72, while the Het/Hom for the newly identified variants (> 1% AF) outside the strict mask is 2.08 across all samples. We then examined possible relatedness in our dataset using the genome option in plink-v1.9 (Chang et al., 2015) calculated using a linkage disequilibrium pruned set of 834k biallelic SNVs (minor allele frequency > 5%, missingness < 2%, –indep-pairwise 50 5 0.5). We excluded one sample from a pair showing PI_HAT > 0.15, leaving 136 samples for analysis.

#### Ancient DNA dataset

We merged our new data with published ancient data extracted from the Allen Ancient DNA Resource curated dataset v44.3; https://reich.hms.harvard.edu/allen-ancient-dna-resource-aadr-downloadable-genotypes-present-day-and-ancient-dna-data). Samples used in our analysis were published in Agranat-Tamir et al., 2020, Allentoft et al., 2015; Antonio et al., 2019; de Barros Damgaard et al., 2018; Feldman et al., 2019; Fregel et al., 2018; Fu et al., 2014; Gamba et al., 2014; Harney et al., 2018; Jones et al., 2015; Lazaridis et al., 2017, 2016; Lipson et al., 2017; Gallego Llorente et al., 2015; Mathieson et al., 2015, 2018; Narasimhan et al., 2019; Olalde et al., 2018, 2019; Prendergast et al., 2019; van de Loosdrecht et al., 2018; Schuenemann et al., 2017; Villalba-Mouco et al., 2019; Mittnik et al., 2018; Günther et al., 2015. We also extracted modern individuals from worldwide populations genotyped on the Human Origins array (Patterson et al., 2012, Lazaridis et al., 2014, Lazaridis et al., 2016). We added ancient and modern Levantines (Haber et al., 2017; Haber et al., 2019), modern Ethiopians (Pagani et al., 2015), and modern Qataris (Rodriguez-Flores et al., 2016). We converted the coordinates of the published data to the human genome assembly GRCh38 using CrossMap (Zhao et al., 2014) and used Graphtyper (Eggertsson et al., 2017) to genotype our samples for positions found in the ancient DNA data using default parameters and set GQ < 20 to missing. We used bcftools option fixref to fix strand orientation and then merged the datasets using bcftools merge and filtering for triallelilic SNPs and sites that were outside the accessibility mask defined in Bergström et al., 2020. The final dataset included 1.09M SNPs (for the 1240k) and 579k SNVs (for the Human Origin array variants). For the LD-decay tests we excluded the samples genotyped on the Human Origin array. We add the suffix “.HO” to the names of the published modern Middle Eastern populations to differentiate them from our new samples.

#### Y chromosome analysis

Y chromosome data of 79 males from the current study were complemented by 46 samples from Haber et al., 2019 and 1208 samples from Hallast et al. (2020). Genotype calling, filtering and Y haplogroup prediction are described in detail in Hallast et al. (2020). Additionally, 11 samples with > 4% of missing data from Haber et al., 2019 were removed from the final analysis. After filtering a total of 1322 samples and 10,194,410 sites remained, including 90,810 variant sites.

All 79 males from the current study, 35 males from Haber et al., 2019 and 332 selected informative males in the context of the study from Hallast et al. (2020) were used to estimate the ages of internal nodes in the Y phylogeny using the coalescence-based method implemented in BEAST (v1.8.4, Drummond and Rambaut 2007). This dataset of 446 samples contained 10,194,410 sites, including 49,728 variant sites. A starting maximum likelihood phylogenetic tree for BEAST was constructed with RAxML (v8.2.10, Stamatakis 2014) with the GTRGAMMA substitution model using variant sites. Markov chain Monte Carlo samples were based on 277 million iterations, logging every 1,000 iterations and the first 10% of iterations discarded as burn-in. LogCombiner was used to combine 20 independent runs. The HKY substitution model accounting for site heterogeneity (gamma), a constant-sized coalescent tree prior and strict clock with a substitution rate of 0.76 x10^−9^ (95% confidence interval: 0.67x10^−9^ to 0.86x10^−9^) single nucleotide mutations per bp per year (Fu et al., 2014) was used. A prior with a normal distribution based on the 95% confidence interval of the substitution rate was applied. Only the variant sites were used, but the number of invariant sites was defined in the BEAST xml file. A summary tree was produced using TreeAnnotator (v1.8.1) and visualized with the FigTree software (Figure S2; http://tree.bio.ed.ac.uk/software/figtree/).

#### PHEWAS and eQTL analysis

We used the Phewas search option in the GWAS atlas (Watanabe et al., 2019) and Gene Atlas (Canela-Xandri et al., 2018) to look for trait associations with variants that show evidence of selection. We used the GTEx portal (GTEx Analysis Release V8; The GTEx Consortium, 2020) to look for eQTL associations.

### Quantification and statistical analysis

#### Chromopainter/fineSTRUCTURE and GLOBETROTTER

We combined our dataset with published modern global populations extracted from the Allen Ancient DNA Resource curated dataset v44.3 (https://reich.hms.harvard.edu/allen-ancient-dna-resource-aadr-downloadable-genotypes-present-day-and-ancient-dna-data). Samples from the curated dataset analyzed in this study were published in Patterson et al. (2012) and Lazaridis et al. (2016). We also added Ethiopian populations (Pagani et al., 2015). Variants were lifted over to GRCh38 using CrossMap (Zhao et al., 2014). We used a minor allele frequency filter > 5%, excluded variants with < 2% missingness and variants that show departure from Hardy-Weinberg equilibrium (< 1e-10). To avoid bias from different phasing methods, as advised by the authors of the Chromopainter/FineSTRUCTURE software, we discarded for this specific test the physical phasing from our samples and phased the merged dataset with Eagle v2.4.1 (Loh et al., 2016) using the 1000 Genomes Project phase 3 panel (1000 Genomes Project Consortium et al., 2015). We then ran the Chromopainter/FineSTRUCTURE pipeline v4.1.1 using ∼400K variants (Lawson et al., 2012). We initially ran the pipeline on a total of 517 samples to identify homogeneous populations. We also ran the pipeline on a limited set of 303 Middle Eastern samples to look at the region in more detail (Figure 1C), here we included the EmiratiA and SaudiA from the Emirati and Saudi populations. We ran the pipeline twice for each dataset to assess variability and found generally consistent results. We divided some self-labeled populations into relatively homogeneous subpopulations: A-D, for Emiratis and A-B for Saudis, based on the haplotype clustering and single-variant model-based clustering (Figure 1B).

To date and investigate potential sources of admixture, we used fastGLOBETROTTER (Figure S1), a newer implementation of GLOBETROTTER which was originally described in Hellenthal et al. (2014), using the parameters (prop.ind: 1, bootstrap.date.ind: 1, null.ind: 1, with all remaining parameters default) We chose donor populations defined by the initial fineSTRUCTURE results and in some cases combined some populations that clustered together. We ran ChromopainterV2 based on a set of the following donor populations (“_” illustrates that populations were combined): Assyrian_Armenian_Georgian, Bantu_Kenya_Luhya_Luo, BantuSA, Bulgaria_Albania, Chechen_Kum_Lez, Esan_Yoruba, Gumuz, Iranian, Iranian.Bandari, Kalash, Khomani_San, Kyrgyz, Lebanese, Makrani_Brahui_Balochi, Malta_Sicily, Mbuti_Biaka, Mende_Mandenka, Pathan_Sindhi, Punjabi_Burusho, Sardinian, Uzbek_Turkmen.

#### MALDER

We tested for admixture using modern samples as references with MALDER v1.0 (Loh et al., 2013; Pickrell et al., 2014). We included ∼580k variants using 6 references: (Luhya in Webuye, Kenya (LWK.SG);Yoruba;Druze;Iranian;Indian Telugu in the UK (ITU.SG); Punjabi in Lahore, Pakistan (PJL.SG) setting mindis: 0.005 and using a generation time of 29 years (Table S1). MALDER and GLOBETROTTER provide generally similar admixture dates.

We also used MALDER v1.0 (Loh et al., 2013; Pickrell et al., 2014) with parameters mindis: 0.005, binsize: 0.0005 and a generation time of 29 years to estimate admixture time related to ancient Iranians from decay of LD (Table S2). We tested our populations using weights from ancient Levantines and ancient Iranians: Levant_N, Levant_ChL, GanjDareh_N, and Tepe_Hissar_ChL. We similarly tested admixture in East Africans but replaced the ancient Levantines references with Gumuz and Yoruba.

#### Principal component analysis and model-based clustering

We computed a PCA using smartpca v16000 from the EIGENSOFT package (Patterson et al., 2006) with parameters numoutlieriter: 0, lsqproject: YES, autoshrink: YES and using only variation in modern populations selected to represent genetic diversity in Central/South Asia, the Middle East, and Europe (Figures 1D and S1).

We ran DyStruct (Figure 1B; Joseph and Pe’er, 2019) in an unsupervised mode from K = 6 to K = 20 using ∼88,000 transversions in our dataset which we randomly subsetted to ≤ 10 individuals per modern population and ≤ 20 individuals per ancient population. We ran DyStruct with default arguments across nine time points binned as follows (in years ago): 14,500-10,000; 10,000-8000; 8000-6000; 6000-5200; 5200-5000; 5000-3000; 3000-1400;1400-200; and present-day.

#### f4 statistics, qpAdm and qpGraph

From the ADMIXTOOLS package (Patterson et al., 2012) we used qpDstat v755 with parameter f4mode: YES to test the genetic contrast between North and South of the Middle East (Figure 1E) and to assess the amount of Neanderthal and Basal Eurasian ancestry. We used *qpAdm* v810 with option allsnps: YES to estimate ancestry proportions in our samples and used *qpGraph* v6450 to draw phylogenetic models that explain the formation of populations in the Middle East.

#### Demographic history

We leveraged the physical-phasing in our dataset using RELATE v1.1 (Speidel et al., 2019) to examine effective population size and separation history. We limited analysis to regions within the genome accessibility mask described in Bergström et al., 2020 and set unphased variants to missing (i.e., excluded from analysis). We then converted phased VCFs to the haps/sample file format using the RelateFileFormats script (part of the Relate package) and prepared the input files using PrepareInputFiles.sh. We supplied the human ancestor sequences downloaded from ftp://ftp.ensembl.org/pub/release-100/fasta/ancestral_alleles/ to polarize variants as ancestral or derived. We then ran Relate with with options -m 1.25e-8 -N 30000 using the HapMap genetic map supplied with Eaglev2.4.1 (genetic_map_hg38_withX.txt.gz) then used the output in the EstimatePopulationSize.sh script with options -m 1.25e-8 –years_per_gen 29.

We were concerned that the decrease in population size we find around 4-5kya could be a result on recent consanguinity, which is common in the Middle East. To test this, we repeated the population size analysis, first including samples with high total sum of runs of homozygosity (sROH > 50 Mb; minimum ROH size of 1Mb), second including samples with relatively low sROH (< 50 Mb) and third by choosing one haplotype per individual, instead of two haplotypes. This will remove the effect of recent consanguinity, as we also removed any related samples as discussed in the ‘Samples Processing and Quality Control’ section. We show the results of these tests in Figure S4C. Including samples with high total of ROH amplifies the population reduction in the past 4ky and then a modest recovery in the last 1ky is observed. Samples with relatively lower ROH show a similar history, but the recent decrease in size is more attenuated and a stronger recovery is observed in the last 1ky. The single haplotype curves show that the recent recovery is slightly older and begins at 2kya and results in a larger increase in population size in comparison to the previous two curves. The second bottleneck appears in all curves. We also repeated the analysis on single haplotypes separately for both Levantines/Iraqis and Arabians and find similar results (Figure 4A). Figure 4A in the main text refers to the single haplotype analysis and included EmiratiA and SaudiA, for the other Emirati and Saudi subpopulations we show them in Figure S4E. For the separation history analysis Figure 4C we used diploid samples with the low sROH.

As we find a divergence in population size between Arabia and the Levant before 10kya, i.e., before the Neolithic era, we reran the analysis using another method (MSMC2 v2.1.1; first described in Schiffles and Durbin, 2014 with later version MSMC2 published in Wang et al., 2020) to check for concordance with the results from Relate. We used 4 haplotypes from 4 individuals (1 haplotype per individual) per population. The results from MSMC2 agree with Relate, the divergence in size starts before 10kya (Figure S4A).

For the separation history analysis with global populations, we downloaded the Mbuti, Sardinian, and Han Chinese physically-phased samples from the HGDP (2 samples per population, Bergström et al., 2020). Since the published data used an older version of Long Ranger, we recalled the samples using v.2.2.2 to be consistent with our dataset. We filtered the VCFs as described above for our dataset. We used MSMC2 v2.1.1 to infer split times between our populations and the HGDP samples using 8 haplotypes for each comparison (4 haplotypes from each population). MSMC2 was run using the –skipAmbiguous option, to calculate coalescent rates within and between populations, restricted to the genome accessibility mask described in Bergström et al., 2020. We used a generation time of 29 years and a mutation rate of 1.25e-8 to scale the results. We then used MSMC-IM (Wang et al., 2020) on the output of the previous step to infer migration rates from coalescent rates using default parameters (Figure S5). We excluded from analysis migration rates when the cumulative migration probability reached over 0.999, as suggested by the authors.

#### Archaic admixture

We used IBDMix (Chen et al., 2020) to call Neanderthal segments in a merged dataset of our samples with the HGDP. We downloaded the high coverage Altai Neanderthal (Prüfer et al., 2014) and Denisova (Meyer et al., 2012) VCFs from http://cdna.eva.mpg.de/neandertal/Vindija/VCF/. We excluded modern populations with less than 10 samples as suggested in Chen et al. (2020) and followed the filtering steps they previously described: We removed sites that lie within segmental duplications downloaded from (http://hgdownload.cse.ucsc.edu/goldenPath/hg38/database/genomicSuperDups.txt.gz), removed variants that are CpG, restricted analysis to the previously described HGDP accessibility mask, in addition to the archaic genome accessibility masks downloaded from https://bioinf.eva.mpg.de/altai_minimal_filters/. We removed singletons, variants that deviate from Hardy-Weinberg equilibrium (< 1e-10), show excessive heterozygosity (< 1e-8), and only included biallelic SNVs. As performed in Chen et al. (2020), we also ran IBDMix to identify ‘Denisovan’ segments in African populations and masked these regions in non-Africans as they are likely to be enriched for incomplete lineage sorting. We filtered the remaining segments using a minimum size threshold of 50kb and LOD score higher than 4.

We also ran Sprime (Browning et al., 2018) on a similar dataset as above but without excluding CpG sites, regions of segmental duplications and the archaic genome accessibility masks (Figure S6). As Sprime requires non-missing genotypes, we removed variants that were > 5% missing and imputed the remaining missing variants using Eaglev2.4.1 (Loh et al., 2016). We set all non-Middle Eastern samples from the HGDP as outgroup (768 samples) and ran Sprime for each Arabian population. We filtered the output using a score threshold of 150,000.

We applied MSMC2 using 4 haplotypes (2 diploid samples) to examine the separation history between our populations and the high coverage Vindija Neanderthal (Prüfer et al., 2014) as we did in our previous study (Bergström et al., 2020). Briefly, this analysis exploits the fact that the Vindija Neanderthal shows extremely low heterozygosity, which renders much of the genome homozygous and essentially phased. We used the –skipAmbiguous option to exclude sites with unknown phase and ran MSMC2 using the same parameters previously stated.

#### Selection

We used the Relate Selection Test in RELATE v1.1 (Speidel et al., 2019) to look for lineages that spread faster than competing lineages. At every site, all variants are required to be phased to perform this test and our relatively stringent quality control may set some variants as missing (for e.g., if a sample had a duplication at a locus, variants within the region will be set as missing because of our depth filter), so we relaxed the depth filter for this test. In addition, as the 10X linked-read technology phases ∼98% of variants, we used BEAGLEv4.0 (Browning and Browning, 2007) to statistically phase the remaining variants using the gtgl option only for this specific selection test. We set the option usephase = true to take into account the physical-phasing already provided in the VCF*.* We first ran RELATE v1.1 on 272 haplotypes using the same parameters described in the demographic history section. We extracted the genealogies of the Arabian samples and used the output of the previous step as input to the DetectSelection.sh script accounting for the population history of the populations using the option -m 1.25e-8 –years_per_gen 29. From the resulting .sele file, we extracted p values from the column “when_mutation_has_freq2” which tests for evidence of selection over the lifetime of a particular variant. We were conservative and only included variants that show significance at a “genome-wide threshold” of p < 5e-8. This test for significance has been shown to be well calibrated (Speidel et al., 2019), but to even further refine and understand the evolutionary history of the variant we used CLUES (Stern et al., 2019; https://github.com/35ajstern/clues). From the output of Relate above, we ran the SampleBranchLengths.sh script to sample branch lengths from the posterior in order to account for uncertainty. We ran 100 samples (–num_samples 100) using a mutation rate of 1.25e-8 and accounted for the population size history by supplied the .coal files from the previous step. We then ran CLUES (inference.py script) with the option –coal {.coal file} to again account for population size changes. We fine-mapped variants using the likelihood ratio statistic produced by CLUES as suggested by Stern et al. (2019) and focused on variants that show moderate to strong selection (s > 0.005). We used the plot_traj.py script to plot the results.

To test for polygenic selection, we used PALM (Stern et al., 2021; https://github.com/35ajstern/palm). We avoided the use of GWAS summary statistics that were calculated from meta-analysis, due to the potential effect of uncorrected population stratification. We extracted GWAS summary statistics performed on the UK BioBank (UKBB; (Bycroft et al., 2018) downloaded from (https://alkesgroup.broadinstitute.org/UKBB/UKBB_409K/ - (Gazal et al., 2018); https://alkesgroup.broadinstitute.org/UKBB/LTFH/sumstats/ - (Hujoel et al., 2020) ; and from the Neale lab Imputed v3 dataset http://www.nealelab.is/uk-biobank/). These statistics were nominally corrected for population structure using either a family history-based approach, fixed PCs, or a linear mixed model. For each trait investigated, we split the genome into 1,700 approximately independent blocks (Berisa and Pickrell, 2016) and selected the variant with the lowest p value within each block for analysis. We chose to be conservative by only including blocks with variants that were genome-wide significant (p < 5e-8), as potentially uncorrected population structure is not expected to produce such highly significant values. Moreover, the method we use, PALM, has been shown to perform well even with some uncorrected GWAS stratification (Stern et al., 2021). Variants were filtered for minor allele frequency > 5%, Rsq > 0.5, INFO score > 0.8, and excluded indels. For each variant passing the previous thresholds, we sampled branch lengths as done for CLUES and estimated selection likelihoods with the lik.py script using the options–coal {.coal file} to account for population size changes and options –K 1 –kappa 3 to specifically test for selection over the past 2000 years, assuming a generation time of 29 years. We then ran palm.py to test for polygenic selection using the option –B 1000 (number of bootstraps). To further explore the choice of significance threshold, and potential effects of uncorrected population structure (variants passing a higher significance threshold are likely to be less biased by uncorrected population structure), on the results, we repeated the analysis using more stringent significance thresholds (p < 1e-8 and p < 5e-9; which will drop blocks not passing the threshold), and found similar values.

#### Runs of homozygosity analysis

We used plinkv1.9 (Chang et al., 2015) to first filter and prune our dataset using the options: --geno 0.05 --indep-pairwise 50 5 0.5 --maf 0.05 and subsequently identified runs of homozygosity (ROHs) using the option–homozyg with all other options kept as default. We restricted the analysis to the strict mask defined previously. This identifies ROHs ≥ 1 Megabase.

#### Local ancestry deconvolution

We used RFMix v2.03 (Maples et al., 2013) to identify African haplotypes within our dataset. We used 105 samples from the HGDP as references: 41 Druze and 64 Africans. The samples were chosen based on a previous ADMIXTURE run (Bergström et al., 2020) and outliers were excluded (i.e., Druze that show relatively high African ancestry, or Africans that show relatively high Eurasian ancestry). RFMix was run using the option -e 5 for 5 EM iteration steps with all other options set as default.

## Data Availability

Raw read alignments are available from the European Nucleotide Archive (ENA) under study accession number ENA:ERP110713. Phased VCFs are available on ftp://ngs.sanger.ac.uk/production/appg/.

## Supplemental information

Table S1. Testing modern populations for admixture using via MALDER, related to Figure 1 Populations with asterisk (^∗^) have evidence of two pulses.

Table S2. Testing for admixture in modern populations using ancient sources via MALDER, related to Figure 2 Arabian represents both EmiratiA and SaudiA populations combined. Populations with asterisk (^∗^) have evidence of two pulses.

Table S3. Modeling Middle Easterners and East Africans as descending from A, EHG, and Mota, related to Table 1 Showing qpAdm *P value* for rank = 2 and testing with different A. Bold values (p value > 0.05) indicate the model cannot be rejected. We tested if we can model our populations as deriving ancestry from one of the sampled regional Bronze Age populations, and found that the Middle Bronze Age population from Sidon (Sidon_BA) could be a source of ancestry for some modern Levantine and Arabian populations (See also Table S4). We used the following outgroups in the qpAdm test: Ust_Ishim, Kostenki14, Papuan, WHG, CHG, Mbuti, Levant_N and GanjDareh_N.

Table S4. Ancestry proportions from successful models tested in Table S3, related to Table 1 Showing models involving Sidon_BA or Egypt_prePtolemaic as one of the ancestry sources.

Table S5. Population labels of ancient samples, related to Figures 1, 2, and 3 and Tables 1, S2, S3, and S4

Table S6. Number of SNPs included in polygenic selection tests per phenotype, related to Figure 6D
